# Antioxidant-Rich Functional Foods and Exercise: Unlocking Metabolic Health Through Nrf2 and Related Pathways

**DOI:** 10.3390/ijms26031098

**Published:** 2025-01-27

**Authors:** Halina Tkaczenko, Natalia Kurhaluk

**Affiliations:** Institute of Biology, Pomeranian University in Słupsk, Arciszewski St. 22b, 76-200 Słupsk, Poland; halina.tkaczenko@upsl.edu.pl

**Keywords:** Nrf2, PI3K/Akt pathway, Nrf2/Keap1/ARE signalling system, physical activity, antioxidant-rich diet, synergistic effect

## Abstract

This article reviews the synergistic effects of antioxidant-enriched functional foods and exercise in improving metabolic health, focusing on the underlying molecular mechanisms. The review incorporates evidence from PubMed, SCOPUS, Web of Science, PsycINFO, and reference lists of relevant reviews up to 20 December 2024, highlighting the central role of the Nrf2 pathway. As a critical regulator of oxidative stress and metabolic adaptation, Nrf2 mediates the benefits of these interventions. This article presents an innovative approach to understanding the role of Nrf2 in the regulation of oxidative stress and inflammation, highlighting its potential in the prevention and treatment of various diseases, including cancer, neurodegenerative disorders, cardiovascular and pulmonary diseases, diabetes, inflammatory conditions, ageing, and infections such as COVID-19. The novelty of this study is to investigate the synergistic effects of bioactive compounds found in functional foods (such as polyphenols, flavonoids, and vitamins) and exercise-induced oxidative stress on the activation of the Nrf2 pathway. This combined approach reveals their potential to improve insulin sensitivity and lipid metabolism and reduce inflammation, offering a promising strategy for the management of chronic diseases. However, there are significant gaps in current research, particularly regarding the molecular mechanisms underlying the interaction between diet, physical activity, and Nrf2 activation, as well as their long-term effects in different populations, including those with chronic diseases. In addition, the interactions between Nrf2 and other critical signalling pathways, including AMPK, NF-κB, and PI3K/Akt, and their collective contributions to metabolic health are explored. Furthermore, novel biomarkers are presented to assess the impact of these synergistic strategies, such as the NAD^+^/NADH ratio, the GSH ratio, and markers of mitochondrial health. The findings provide valuable insights into how the integration of an antioxidant-rich diet and regular exercise can improve metabolic health by activating Nrf2 and related molecular pathways and represent promising strategies for the prevention and treatment of metabolic disorders. Further studies are needed to fully understand the therapeutic potential of these interventions in diseases related to oxidative stress, such as cardiovascular disease, neurodegenerative disease, diabetes, and cancer.

## 1. Introduction

Metabolic health encompasses the effective functioning of the body’s metabolic systems, including the regulation of blood glucose levels, lipid processing, insulin sensitivity, and the ability to adapt to fluctuations in energy demand [1,2]. Ensuring metabolic health is crucial for the prevention and management of chronic conditions, such as obesity, type 2 diabetes, and cardiovascular diseases, as demonstrated by Bonilla et al. [3]. These conditions often result from impairments in the body’s metabolic regulation, which can lead to increased oxidative stress, inflammation, and insulin resistance. Consequently, the implementation of strategies to restore and maintain metabolic balance is essential to address these escalating global health issues [4].

Bioactive antioxidant-rich foods and regular physical activity are distinct yet synergistic approaches that have demonstrated potential to improve systemic health [5]. Functional foods containing bioactive compounds, such as polyphenols, flavonoids, and vitamins, are known to reduce oxidative stress, improve mitochondrial function, and regulate inflammatory pathways [6]. Similarly, exercise is widely recognised for its role in improving insulin sensitivity, optimising lipid metabolism, and promoting beneficial cellular and metabolic adaptations [7]. While both interventions are effective individually, their combined implementation may provide enhanced benefits for metabolic health [8].

This article proposes that combining antioxidant-enriched functional foods with exercise offers synergistic benefits by simultaneously addressing oxidative stress and promoting metabolic adaptation at the molecular level. Both approaches activate critical pathways that enhance the body’s ability to manage oxidative damage, optimise energy metabolism, and alleviate chronic inflammation [9,10]. By exploring the interplay between these strategies, researchers may be able to develop more effective interventions to prevent or treat metabolic disorders.

Molecular research provides a basis for understanding how daily dietary and physical activity choices affect health [11]. A central focus is the interplay between oxidative stress and antioxidants [12]. Oxidative stress occurs when free radicals accumulate in the body, leading to cellular damage and contributing to chronic diseases, such as diabetes, cancer, and cardiovascular diseases. Antioxidants found in nutrient-rich foods, such as vitamins C and E, polyphenols, and minerals, neutralise these harmful molecules while supporting DNA repair and cellular protection [13]. Therefore, the translation of scientific knowledge into practical applications has significant potential to improve the quality of life.

Recent studies have highlighted the pivotal role of antioxidant-rich foods in activating Nrf2 pathways, underscoring their importance in mitigating oxidative stress and promoting overall health [14]. Known as a redox-sensitive transcription factor [15], Nrf2 regulates the expression of genes with antioxidant-responsive elements (ARE) in their promoters [16]. In cells, Nrf2 is tightly regulated by the repressor protein Keap1, which serves as a molecular “sensor” for fluctuations in intracellular homeostasis. These components form an integrated redox-sensitive signalling system, termed Nrf2/Keap1/ARE [17]. Evidence suggests that the activity of this system is controlled by multiple regulatory mechanisms that depend on the redox balance within cells [18,19]. Research has shown [20,21] that these mechanisms include transcription, translation, post-translational modifications, and translocation of Nrf2 into the nucleus and its binding to target gene promoters [22].

This research provides valuable insights into how dietary and physical activity choices affect health. In particular, the Nrf2/Keap1/ARE pathway plays a central role in cellular responses to oxidative stress and is recognised as a key factor in ageing, chronic diseases, and general health [23]. By elucidating the influence of lifestyle factors, such as diet and exercise, on the activity of this pathway, the study provides insight into strategies to better manage oxidative stress and improve long-term well-being. The study investigates how antioxidant-rich foods and exercise activate Nrf2 and interact with other key pathways, including AMPK, NF-κB, and PI3K/Akt, to improve metabolic function, reduce oxidative stress, and support mitochondrial health. In addition, the research aims to identify biomarkers to assess the combined effects of these interventions on metabolic balance and their potential for the prevention and treatment of metabolic disorders.

This review aims to provide a clearer understanding of the role of Nrf2 in the regulation of oxidative stress and inflammation, highlighting its importance in the prevention and treatment of a wide range of diseases, including cancer, neurodegenerative disorders, cardiovascular and pulmonary diseases, diabetes, inflammatory conditions, ageing and infections such as COVID-19. The novelty of the review lies in its exploration of how Nrf2 activation is influenced by bioactive compounds found in functional foods, such as polyphenols, flavonoids and vitamins, in addition to exercise-induced oxidative stress. By focusing on the synergistic effects of diet and exercise on oxidative stress, mitochondrial function and metabolic processes, this study highlights their potential to improve insulin sensitivity, improve lipid metabolism and reduce inflammation. This integrated approach offers a novel perspective on the management of diseases associated with oxidative stress and inflammation and provides a comprehensive framework for future research and therapeutic strategies. This review provides a novel perspective by explicitly integrating the effects of bioactive compounds in functional foods and exercise-induced oxidative stress on Nrf2 activation, a combination that has not been thoroughly explored in the existing literature. In contrast to previous studies, this review highlights the synergistic consequences of diet and physical activity on Nrf2 regulation, oxidative stress, mitochondrial function, and metabolic processes, providing a comprehensive framework for understanding their collective impact on metabolic health, including the improvement of insulin response, regulation of lipid metabolism, and reduction of inflammatory markers.

The aim of this study is to investigate the synergistic effects of antioxidant-enriched functional foods and exercise in improving metabolic health. It focuses on understanding the molecular mechanisms underlying these interventions, in particular the role of the Nrf2 pathway, a critical regulator of oxidative stress and metabolic adaptation. This article will focus on the role of Nrf2 and its interaction with Keap1 in regulating redox-sensitive signalling pathways that influence gene expression. Understanding these molecular mechanisms is essential to elucidate how cellular responses to oxidative stress are modulated. In addition, the article highlights the importance of these processes in the broader context of health maintenance and disease prevention.

## 2. Antioxidant-Fortified Functional Foods

Antioxidant-enriched functional foods naturally contain such compounds as food extracts and phytochemical formulations derived from plant sources that help neutralise harmful reactive oxygen species (ROS) and reduce oxidative stress in the body. The role of these foods in combating oxidative stress has been demonstrated in various studies [24,25]. Oxidative stress occurs when there is an imbalance between the production of ROS and the body’s ability to neutralise these reactive species, leading to damage to cells, proteins, lipids, and DNA. Over time, this oxidative damage can contribute to the development of chronic diseases, including such metabolic disorders as obesity, type 2 diabetes, and cardiovascular diseases [4,26]. Functional foods are valued not only for their nutritional content but also for their ability to provide specific health benefits, particularly in terms of disease prevention and maintenance of metabolic balance [27].

Recent studies have identified functional foods that are particularly rich in antioxidants, including fruits, vegetables, nuts, seeds, whole grains, and specific phytochemicals such as polyphenols, flavonoids, and carotenoids [28]. The list of these compounds, known for their health benefits beyond basic nutrition, particularly in disease prevention, continues to grow as new research is being conducted and discoveries are being made. This ongoing exploration of natural and synthetic compounds for therapeutic use highlights the potential of these bioactive compounds. These potent antioxidants help to reduce oxidative stress by neutralising ROS and boosting the body’s antioxidant defences [25]. For example, berries, such as blueberries and strawberries, are rich in anthocyanins [29], which have been linked to the prevention of metabolic syndrome, while green tea contains catechins, such as epigallocatechin gallate (EGCG), which are known to have significant antioxidant and anti-inflammatory properties [30]. These foods are thought to support metabolic processes by improving insulin sensitivity, reducing inflammation, and improving mitochondrial function, all of which are essential for maintaining metabolic balance [5].

**Figure 1:**
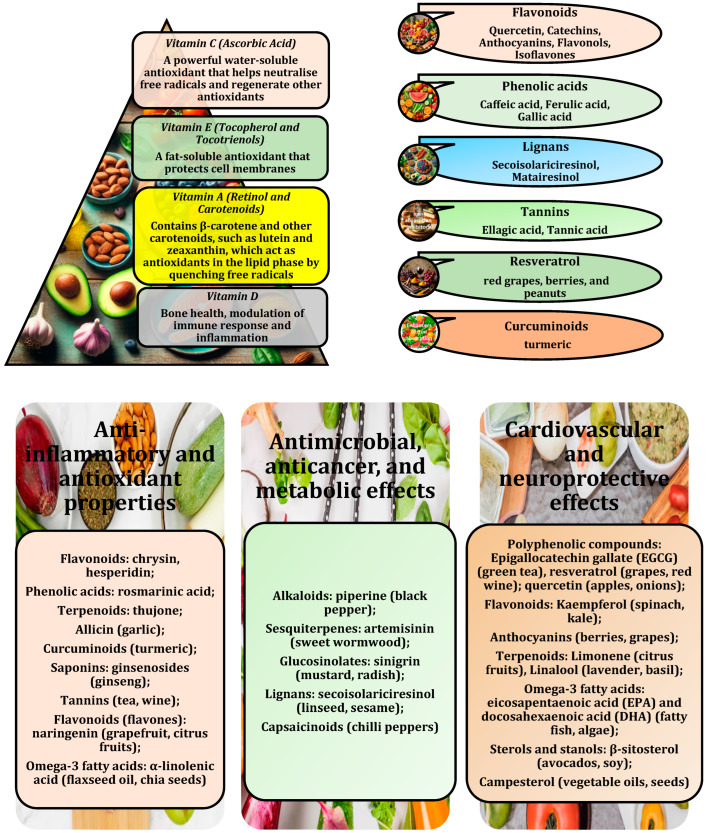
Vitamins with antioxidant properties; some of the most important polyphenolic compounds; compounds with anti-inflammatory, antimicrobial, anticancer, metabolic, cardiovascular, and neuroprotective effects.

Nutrient-dense foods rich in antioxidants contain compounds that help neutralise harmful ROS production, reduce oxidative stress, and support metabolic health [8]. One such category of foods is fruits and berries, which are known for their high antioxidant content [31]. Other antioxidant-rich fruits include oranges, kiwis, and pomegranates, all of which are rich in vitamin C and polyphenolic compounds that enhance the body’s ability to combat oxidative stress [32] (Figure 1).

In addition to fruits, vegetables are another important source of antioxidants, especially green leafy vegetables, as highlighted in a study by Hanna et al. [33]. Vegetables such as spinach, kale, and broccoli are rich in antioxidants, e.g., vitamin C, vitamin E, and carotenoids, including lutein and zeaxanthin [34,35]. These carotenoid isomers play an important role in eye development and function. These compounds are important in reducing oxidative stress and inflammation, both of which are closely associated with such metabolic disorders as obesity and type 2 diabetes [4,26]. In addition, other vegetables, such as tomatoes, contain lycopene, a carotenoid with potent antioxidant properties that can help reduce oxidative stress and improve metabolic markers, e.g., insulin sensitivity. Lycopene also has beneficial effects on serum lipid levels, endothelial dysfunction, inflammation, blood pressure, and overall antioxidant potential [34,35].

Another group of antioxidant-rich foods includes nuts and seeds, which are nutrient-dense and provide a combination of healthy fats and antioxidants [36]. For example, walnuts, almonds, and flaxseeds are rich in vitamin E, polyphenols, and omega-3 fatty acids, all of which help reduce inflammation and oxidative stress [37]. These nuts have been shown to improve lipid profiles and support healthy glucose metabolism. Chia and hemp seeds also contain antioxidants, e.g., polyphenols and flavonoids, which, along with their high fibre and omega-3 content, contribute to overall health by promoting fat metabolism and reducing inflammation, as reported by Marcinek and Krejpcio [38].

Finally, green tea and coffee beverages are rich in potent antioxidants, such as catechins and polyphenols. Green tea in particular is rich in EGCG, which has been shown to increase fat oxidation, support glucose levels, and reduce inflammation [30,39]. Similarly, coffee contains chlorogenic acids, which provide antioxidant and anti-inflammatory benefits, help regulate glucose metabolism, and improve insulin sensitivity, as demonstrated by Tajik et al. [40]. When consumed as part of a balanced diet, these beverages can be a valuable addition to a metabolic health programme due to their high antioxidant content and potential to increase fat-burning and energy expenditure.

The metabolic support role of antioxidant-enriched functional foods goes beyond their ability to reduce oxidative stress. These foods are critical in maintaining a balanced inflammatory response, which is essential for proper insulin signalling and glucose metabolism. Tristan Asensi et al. [41] showed that chronic low-grade inflammation, a hallmark of metabolic disorders, can lead to insulin resistance—a key feature of type 2 diabetes and obesity, especially with the consumption of ultra-processed foods. By incorporating bioactive antioxidant-rich foods into the diet, individuals can help modulate inflammatory pathways and reduce the systemic inflammation that contributes to these conditions. This concept was further supported by a cross-sectional analysis from the Moli-sani study [42]. In addition, antioxidants have been shown to enhance mitochondrial function, improving energy production and metabolic efficiency, both of which are essential components of overall metabolic health [36].

Recent research has shown that the consumption of a variety of nutrient-dense foods rich in antioxidants can be particularly effective in improving physiological markers of health. These include significant improvements in cardiovascular disease risk factors, such as reduced body mass index, total serum cholesterol, blood glucose levels, inflammation, blood pressure, lipid profiles, and body fat composition, with variations observed in those following a vegan diet [43]. While individual antioxidants have demonstrated benefits, consuming a diverse range of antioxidant-rich, nutrient-dense foods provides a broader range of bioactive compounds that work synergistically to support metabolic functions. By incorporating these functional foods into the diet, individuals can take proactive steps to manage and prevent metabolic disorders while promoting overall health and wellness in a holistic manner [44].

Incorporating antioxidant-rich foods into the daily diet can be an effective way to improve health. The focus should be placed not only on consuming adequate amounts but also on the variety of foods to provide the body with a wide range of bioactive compounds. Equally important is bioavailability—the body’s ability to absorb and use nutrients. By strategically combining foods in meals and avoiding factors that reduce nutrient absorption, such as excessive fat or alcohol intake, health benefits can be optimised [32,45].

## 3. Nrf2 as a Master Regulator of Antioxidant Defences

Nrf2 (nuclear factor erythroid 2-related factor 2) is a key transcription factor that serves as a master regulator of the body’s antioxidant defences [46]. It plays an essential role in maintaining cellular redox balance by activating the expression of various antioxidant enzymes that neutralise ROS and prevent oxidative damage [47].

Previous studies [48] have shown that activation of the Keap1/Nrf2 pathway involves disassembly of the Nrf2 complex with the repressor protein Keap1, translocation of the transcription factor into the nucleus, and its dimerisation with the small protein Maf or the transcription factor cJun, together with coactivator proteins. This is followed by an interaction with a cis-regulatory antioxidant-responsive element (ARE) in the promoter regions of redox-sensitive genes [49,50]. Many inducers of the Keap1/Nrf2/ARE signalling system have been discovered [51,52]. Notably, there are two ARE-like sequences in the promoter region of the Nrf2 gene, suggesting that Nrf2 can activate its own expression [53]. As shown in Huang et al. [54], this suggests a positive feedback loop within the Keap1/Nrf2/ARE pathway that significantly increases the sensitivity of the system and the strength of cellular defence mechanisms.

**Figure 2:**
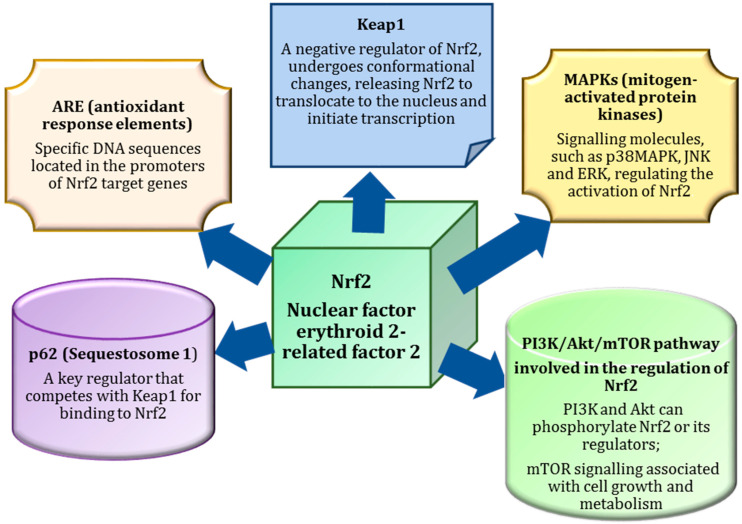
Components of the Nrf2 signalling pathway involved in maintaining cellular homeostasis and protecting cells from damage caused by oxidative stress, thereby contributing to the maintenance of health and the prevention of disease. p62, alternatively known as sequestosome 1, plays an indispensable role in the regulation of Nrf2 by competing with Keap1 for binding. This competitive interaction prevents Keap1-mediated degradation of Nrf2, thereby promoting its activation. In addition, this binding facilitates the transcriptional regulation of antioxidant response elements (ARE), which control the expression of genes involved in cellular defence mechanisms against oxidative stress. In addition, signalling pathways such as MAPKs and the PI3K/Akt/mTOR pathway influence Nrf2 activation, further enhancing its role in cellular survival and stress adaptation, with p62 acting as a critical mediator in this complex regulatory network.

In normal conditions, Nrf2 is bound to Keap1 (Kelch-like ECH-associated protein 1) in the cytoplasm, which promotes its degradation. However, when oxidative stress occurs, ROS or electrophilic molecules modify Keap1, leading to the dissociation of Nrf2. This allows Nrf2 to translocate to the nucleus, where it binds to AREs in the DNA and initiates the transcription of genes involved in detoxification, antioxidant defence, and inflammation regulation. As a result, Nrf2 activation is critical for cellular protection against oxidative damage and plays an important role in maintaining overall cellular health (Figure 2).

The activation of Nrf2 is not only triggered by oxidative stress but can also be modulated by the consumption of antioxidant-rich foods [55]. Many natural compounds found in fruits, vegetables and functional foods, such as sulforaphane (from cruciferous vegetables), curcumin (from turmeric) and catechins (from green tea), have been shown to activate Nrf2 [56]. These compounds interact with the Keap1-Nrf2 pathway either by directly modifying Keap1 or by increasing ROS production in a controlled manner, which subsequently activates Nrf2. This pathway increases the body’s antioxidant capacity, improves detoxification processes and supports metabolic health. By consuming foods rich in these bioactive compounds, individuals can boost Nrf2 activity and strengthen their endogenous defence systems, helping to mitigate oxidative stress and its associated effects on cellular metabolism [18,19].

The activation of Nrf2 plays a critical role in enhancing mitochondrial biogenesis, a process essential for optimising cellular energy production during exercise [57]. When activated in response to exercise-induced ROS, Nrf2 upregulates the expression of several key genes involved in mitochondrial function, including PGC-1α (peroxisome proliferator-activated receptor gamma coactivator 1-alpha) [58]. PGC-1α is a master regulator of mitochondrial biogenesis and plays a crucial role in the formation of new mitochondria in cells, particularly in skeletal muscle. This leads to an increase in the number of mitochondria, which improves the cell’s ability to generate ATP via oxidative phosphorylation [59].

In addition, increased mitochondrial biogenesis increases the efficiency of energy production, allowing better endurance and adaptation to exercise [60]. As suggested by Islam et al. [58], dietary activation of specific pathways may provide greater mechanistic insight into exercise-induced mitochondrial biogenesis in human skeletal muscle, particularly into exercise-induced increases in mitochondrial content and respiratory function. In addition, Nrf2-induced mitochondrial biogenesis helps cells cope with oxidative stress by providing more mitochondrial “powerhouses” [61] that neutralise ROS and prevent damage to cellular structures.

AMP-activated protein kinase (AMPK) has been shown to be a key cellular energy sensor [62] that plays a critical role in regulating energy metabolism during exercise and dietary interventions. AMPK is activated when the AMP/ATP ratio increases, typically during exercise or when cells are under energy stress. Once activated, AMPK promotes glucose and fatty acid uptake, enhances mitochondrial biogenesis, and stimulates oxidative metabolism, ensuring that energy production is matched to cellular needs [63].

AMPK also interacts with the Nrf2 pathway, acting as a signalling hub between energy regulation and antioxidant responses. AMPK activation has been shown to enhance Nrf2 activity by increasing the expression of antioxidant enzymes, thereby contributing to cellular protection against oxidative damage. This cross-talk between AMPK and Nrf2 supports metabolic health by optimising both energy use and antioxidant defences, particularly during physical activity and under oxidative stress [64]. Later research suggests that the synergy between AMPK and Nrf2 helps improve metabolic efficiency and enhances the body’s ability to cope with exercise-induced stress [65,66].

Nrf2 is a key regulator of antioxidant defences, which are essential for maintaining cellular redox homeostasis. Its activation effectively attenuates oxidative stress, highlighting its role in protecting against the development of chronic diseases [67]. Therefore, strategies that increase Nrf2 activity, such as bioactive antioxidant-containing foods and exercise, either alone or in combination, have significant therapeutic potential for disease prevention and treatment [68].

## 4. Antioxidant-Enriched Functional Foods for Disease Prevention

Numerous analyses consistently show that antioxidant-enriched functional foods have emerged as a promising strategy for disease prevention, capitalising on their ability to neutralise oxidative stress and support overall health through bioactive compounds [29,69,70]. However, the long-term effects of supplementation with bioactive antioxidant-containing foods in humans and animals, as well as their broader potential in combating neurodegenerative diseases, have yet to be fully established.

Importantly, many environmental factors play a critical role in age-related cognitive decline and memory impairment associated with Alzheimer’s disease (AD) and other neurodegenerative disorders [71,72]. Multiple sclerosis (MS), a chronic inflammatory neurodegenerative disease, is characterised by demyelination, astrogliosis, axonal degeneration, and sclerotic plaques. Several NRF2-activating compounds, including the approved drug dimethyl fumarate (DMF, Tecfidera), have shown beneficial effects in models of relapsing-remitting MS [73,74]. Notably, loss of Nrf2 is associated with a more severe disease course, including increased glial activation, spinal cord damage, axonal degeneration, and elevated levels of pro-inflammatory cytokines [75]. The main pathological features of Alzheimer’s disease include intracellular neurofibrillary tangles composed of tau protein and extracellular β-amyloid plaques [71]. Nrf2 expression has been shown to be downregulated in Alzheimer’s disease, as demonstrated by a meta-analysis of microarray datasets that identified 31 downregulated genes containing the ARE consensus sequence bound by Nrf2. These genes are part of a negatively regulated antioxidant defence system in AD patients, as shown in a study by Wang et al. [76].

Another therapeutic agent based on Nrf2 activators is sulforaphane (SFN), an isothiocyanate found in cruciferous vegetables, such as broccoli, Brussels sprouts, and cauliflower. SFN activates Nrf2 by direct electrophilic modification of cysteine residues in Keap1, as shown by Takaya et al. [77] and Saito et al. [78]. Its ability to cross the blood-brain barrier makes SFN particularly relevant for protection against neurodegenerative disorders, as shown in various mouse models. In acute brain injury, SFN has been shown to protect against hypoxic-ischemic injury in rats by reducing infarct size and activating Nrf2 and heme oxygenase-1 [79]. The significant influence of diet on epigenetics has also been highlighted, with potential benefits for improving health outcomes. This effect, known as epigenetic inheritance, can even be passed down through generations, as reported earlier by Fitz-James and Cavalli [80].

Compounds that activate the Nrf2-dependent signalling pathway are diverse, but a common feature is their ability to modify the sulphhydryl groups of cysteine residues. Consequently, research has focused on the interaction between the polycysteine sensor Keap1 and Nrf2, which is essential for the transcriptional activity of Nrf2 [78,81]. Furthermore, numerous studies have shown that in addition to ROS-mediated modification of the Keap1 inhibitor protein, ARE inducers also affect signalling cascades involved in the regulation of Nrf2. For example, a study by Gjyshi et al. [82] showed that Nrf2 activation may depend on the autophagic protein sequestosome-1 (SQSTM1/p62) rather than oxidative stress, particularly in the pathogenesis of viral infections and tumourigenesis.

We analysed popular therapeutic agents based on Nrf2 activators, including both natural and synthetic compounds, and summarised the data in Table 1, which highlights selected studies on the impact of Nrf2 activation and functional foods on specific diseases. Among natural activators, soy isoflavones are known to enhance antioxidant defences and reduce inflammatory cytokines. Sulforaphane from cruciferous vegetables protects against neurodegeneration and hypoxic damage, resveratrol prevents ischaemic injury, α-lipoic acid activates mitochondrial biogenesis and protects brain cells, and curcumin reduces ROS levels and is used to treat metabolic disorders [24,25,28]. The synthetic Nrf2 activator dimethyl fumarate (DMF) has been used in the treatment of multiple sclerosis and has been shown to have neuroprotective properties [83].

These studies highlight the role of Nrf2 in regulating oxidative stress and inflammation and demonstrate its therapeutic potential in such conditions as cardiovascular diseases, diabetes, and neurodegenerative disorders. The table provides a comprehensive summary of dietary interventions, targeted diseases, and molecular findings related to Nrf2 pathways.

## 5. Nrf2 and Diseases

The role of Nrf2 has been demonstrated in several diseases associated with impaired macrophage function [87,93]. In certain conditions, the phagocytic activity of macrophages is directly dependent on Nrf2 activity, making them more effective in fighting bacterial infections during the acute phase of inflammation [94]. However, in such inflammatory diseases as atherosclerosis, Nrf2 can stimulate both classical and alternative macrophage activation. The increased production of activated oxygen metabolites, a hallmark of pro-inflammatory polarisation, triggers the Keap1/Nrf2/ARE pathway and, in certain conditions, induces anti-inflammatory ARE signalling [95]. This may be related to the conflicting data on the involvement of Nrf2-dependent processes in macrophage phenotype modification and their coordination with other redox-sensitive signalling systems that regulate inflammation through transcription such factors as NF-κB, PPARγ, AP-1, and others [18,19]. Early reports by Tan et al. [96] suggest that redox signalling influences macrophage polarisation, with distinct roles for M1 and M2 macrophages in the tissue environment, providing insight into why certain phenotypes exhibit higher ROS levels.

The understanding of the mechanisms involved in Nrf2-dependent processes continues to expand. Malhotra et al. [97] analysed an in vivo mouse model using a unique Nrf2 ChIP-Seq dataset and revealed significant enrichment for Nrf2 binding motifs. By integrating ChIP-Seq with microarray data, they identified 645 basal and 654 inducible direct target genes of Nrf2, with 244 genes overlapping between the two groups. The study found that the basal and inducible programmes differed in their involvement in pathways regulating stress responses and cell proliferation. The authors concluded that Nrf2 is a central regulator of the global stress response and a key player in cell survival [97].

Malhotra et al. [97] have shown that Nrf2 in its transcriptionally active form binds to the ARE sequence present in the promoters of genes encoding enzymes involved in antioxidant defence and detoxification of various xenobiotics. This activation of the Keap1/Nrf2/ARE system is critical for macrophage survival, as shown by Crook-McMahon et al. [22]. In addition, several studies have shown that this activation triggers a shift in macrophages from a pro-inflammatory to an anti-inflammatory state, thereby initiating repair processes [98,99].

Nrf2 activators, including both food additives and pharmaceuticals, have shown potential in the prevention of neurodegenerative diseases and oxidative stress-induced damage. For example, t-butylhydroquinone (tBHQ), a synthetic food antioxidant used to prevent oxidative damage to oils and fats, is a well-known Nrf2 activator [100]. However, tBHQ also exhibits dual effects, including chemoprotective and carcinogenic properties, which have been linked to such mechanisms as the formation of reactive GSH conjugates, generation of reactive species, induction of CYP1A1, activation of caspases, and reduction of GSH and ATP levels [100]. Studies have shown that tBHQ reduces neurotoxicity, decreases β-amyloid accumulation in NT2N neurons, and protects against Aβ-induced cell death in rats [101].

Bouvier et al. [20] have shown that the Keap1-Nrf2 pathway plays a key role in stress resistance mechanisms that are critical in the development of depressive disorders. In a rat model of social defeat, the authors demonstrated a link between the development of susceptibility to stressors and persistent oxidative stress, reduced levels of brain-derived neurotrophic factor (BDNF), and inhibition of Nrf2 translocation to the nucleus. This inhibition blocks the activation of antioxidant enzymes, thereby disrupting oxidative homeostasis.

Another study provided compelling evidence for the role of Nrf2 as a tumour suppressor, leading to the idea of targeting Nrf2 activators for cancer prevention [102]. However, this view shifted when it became clear that dysregulated Nrf2 activity could contribute to cancer progression. Zhang et al. [103] found that the Keap1-Nrf2 pathway regulates adaptive homeostasis through such inhibitors as Bach1 and c-Myc, which normally turn off Nrf2 activation. With age, the levels of Bach1 and c-Myc increase, leading to a decrease in Nrf2 signalling and its protective effects. This age-related increase may represent a strategy to reduce cancer risk by limiting Nrf2 activity, suggesting a balance between maintaining homeostasis and minimising cancer susceptibility in older individuals.

In cancer cells, elevated levels of Nrf2 contribute to a protective environment that shields cells from oxidative stress, chemotherapy and radiation, making cancer difficult to treat [104]. Although Nrf2 activation plays a protective role against various insults and diseases, sustained activation has been linked to the progression of lung, breast, head and neck, ovarian and endometrial cancers [105]. The dual role of Nrf2 in cancer is a complex phenomenon that depends on the stage of cancer progression. In the early stages of cancer, Nrf2 activation provides a protective response to oxidative stress, helping to prevent DNA damage and cell death [104,105]. However, in established cancers, prolonged activation of Nrf2 promotes tumour growth by upregulating pro-survival genes, supporting metabolic reprogramming and preventing apoptosis [106]. In addition, Nrf2 has been implicated in increasing cancer cell resistance to chemotherapy and radiation, and in facilitating inflammation-induced carcinogenesis. This dual function highlights the need for a nuanced approach to targeting Nrf2 for cancer therapy, as its effects can vary significantly at different stages of cancer development. This dual role of Nrf2 highlights the complexity of its function in cancer biology [106].

Research has identified several mechanisms that lead to constitutive activation of the Nrf2 pathway in cancer cells [107]. These include somatic mutations in *KEAP1* or its binding domain, epigenetic silencing of Keap1 [108], and post-translational modifications of Keap1 cysteines, e.g., succinylation, together with the accumulation of disruptor proteins, such as p62, which dissociate the Nrf2-Keap1 complex [21]. In addition, the K-Ras, B-Raf, and c-Myc oncogenes can transcriptionally induce Nrf2, and miRNAs can target NFE2L2 or the 3′ UTR of KEAP1 mRNA [69].

## 6. Pathogenesis of SARS-CoV-2 and Nrf2

It should be noted that although the aetiological agent of the disease, known as “COVID-19”, is primarily responsible for causing respiratory disease, there is also evidence that it can affect a variety of non-respiratory organs [109,110,111,112,113]. A comprehensive review of the current literature indicates that following the SARS-CoV-2 pandemic, numerous studies have investigated the interplay between oxidative stress and inflammation due to their association with severe complications, underscoring the systemic nature of the disease and its complex pathophysiology [109,110]. Oxidative stress plays a central role in the pathogenesis of SARS-CoV-2 by exacerbating inflammation and causing tissue damage [111]. The virus activates enzymes such as NADPH oxidases, leading to excessive production of ROS. These molecules damage lipids, proteins and DNA, disrupt cellular functions and increase inflammation by activating pathways such as NF-κB. In addition, SARS-CoV-2 impairs the body’s antioxidant defences, including glutathione-related systems, creating a feedback loop of oxidative stress and inflammation. This vicious cycle contributes to severe complications, such as acute respiratory distress syndrome [112].

Therapeutic approaches targeting this condition have been proposed, focusing on the activation of the transcription factor Nrf2, which supports cellular resilience under oxidative and electrophilic stress [113]. As reported by Zinovkin and Grebenchikov [114], the cytokine storm seen in SARS-CoV-2 and other diseases is closely linked to increased ROS production by immune cells, which drives oxidative stress. In addition, research by Mpekoulis et al. [115] shows that virus-induced tissue hypoxia enhances free radical processes. These researchers found altered expression of genes involved in catecholamine biosynthesis and metabolism in blood samples from hospitalised patients and in cultured cells. SARS-CoV-2 infection was shown to suppress dopamine synthesis, partly due to the hypoxia-like conditions induced by the virus.

As previously reported, hypoxia induced by viral infection disrupts the structure and function of mitochondrial DNA, triggering changes in the components of the mitochondrial respiratory chain and leading to increased levels of superoxide anion radicals [116]. This process is further enhanced by endonuclease activity, which is stimulated by elevated intracellular calcium levels during oxidative stress, particularly in the cardiovascular system [117]. As Nrf2 regulates the expression of genes involved in antioxidant responses, redox homeostasis, mitochondrial biogenesis, and other protective mechanisms, its activation is considered a promising therapeutic approach to counteract the effects of viral infections by boosting antioxidant defences [118]. However, SARS-CoV-2 can inhibit Nrf2 activation through its viral proteins, thereby weakening the cell’s ability to cope with oxidative stress, which in turn increases inflammation and tissue damage, contributing to severe COVID-19 outcomes [110].

Therapeutic strategies focusing on the transcriptional activation of Nrf2 have been outlined in several studies [119,120,121]. In normal conditions, Nrf2 is repressed by Keap1. However, during oxidative stress (e.g., due to excessive ROS), Nrf2 is released and translocates to the nucleus, where it promotes the expression of antioxidant defence genes, such as glutathione reductase and superoxide dismutase [17]. This activation provides cellular protection by reducing inflammation. For example, Nrf2 increases the expression of haem oxygenase-1 (HO-1), which degrades pro-inflammatory free haem and produces anti-inflammatory compounds such as carbon monoxide and bilirubin. It also enhances the activity of NAD(P)H quinone oxidoreductase (NQO1), an enzyme with antioxidant functions [122]. Nrf2 also plays a key role in the synthesis of glutathione, an important cellular antioxidant [67].

In addition to its role in antioxidant responses, Nrf2 acts as a transcriptional repressor reducing the expression of mRNA and protein for pro-inflammatory IL-1β, IL-6, and TNF cytokines in macrophages [123]. The protective effects of Nrf2 activation in various inflammatory responses have been demonstrated in numerous studies in epithelial cells, vascular endothelium, and other systems [124,125]. These findings highlight the potential for Nrf2-targeted therapies to attenuate oxidative stress and inflammation associated with viral infection.

A substantial body of evidence highlights the potential health benefits of natural compounds that activate Nrf2 by reducing oxidative stress and inflammation during SARS-CoV-2 infection. Such substances as sulforaphane, curcumin, resveratrol, genistein, and green tea catechins have shown efficacy in various studies [126,127]. Among these, sulforaphane, derived from broccoli, has been shown to enhance Nrf2 activity and increase levels of antioxidants, such as glutathione [128]. Similarly, research suggests that curcumin, found in turmeric, not only activates Nrf2 but also helps to modulate inflammation [110].

Resveratrol, found in grapes and red wine, has been reported to improve mitochondrial function through Nrf2 pathways [129]. Genistein, a soy isoflavone, protects against oxidative damage by increasing Nrf2 activity, while green tea catechins, particularly epigallocatechin gallate (EGCG), have potent antioxidant effects and activate Nrf2 [130]. These functional food compounds may help alleviate the oxidative stress and cytokine storms induced by SARS-CoV-2 [114].

In addition, numerous studies highlight the role of quercetin, a plant flavonoid, as a potent Nrf2 activator with anti-inflammatory properties [131]. Other compounds such as apigenin (found in parsley and chamomile) and baicalin (derived from skullcap root) also support Nrf2-related pathways [132]. Baicalin, a flavonoid found in several plant species, has been shown to reduce oxidative stress and inflammation. However, it is less emphasised in mainstream antioxidant research. The inclusion of such compounds would not only serve to diversify the range of antioxidants covered by research but would also facilitate a deeper understanding of their therapeutic potential in a range of health conditions [132]. Adding these natural compounds to standard treatments may provide additional protection against viral damage, reduce inflammation, and improve recovery in COVID-19 patients.

## 7. Exercise as a Natural Catalyst for Metabolic Resilience

Recent research has highlighted the important role of antioxidant-rich foods and physical activity in managing oxidative stress and enhancing cellular defences [8,11]. Antioxidant-rich foods provide essential nutrients that activate Nrf2 and mitigate oxidative damage, while regular physical activity complements this by amplifying Nrf2 signalling and boosting the body’s natural antioxidant defences, promoting overall health and resilience [133].

Physical activity has a profound effect on metabolic health, influencing such processes as regulation of glucose levels, lipid metabolism, and mitochondrial biogenesis [26]. Studies show that regular exercise improves the body’s ability to control blood glucose levels by improving insulin sensitivity, which allows more efficient glucose uptake by cells [134,135]. This is particularly important in the prevention and management of insulin resistance, a hallmark of metabolic disorders, such as type 2 diabetes.

In addition, physical activity supports lipid metabolism by increasing fatty acid oxidation, reducing visceral fat and improving lipid profiles. It also stimulates mitochondrial biogenesis, increasing cellular energy production and overall metabolic efficiency [7]. As a result, regular exercise not only supports weight management but also optimises energy use, contributing to metabolic resilience and reducing the risk of metabolic disorders [23,136,137].

Recent studies have shown that ROS and nitric oxide (NO) play a key role in regulating the expression of nuclear factor erythroid 2-related factor 2 (NFE2L2) in the skeletal muscle during exercise [57]. NFE2L2 is essential for the exercise-induced upregulation of genes involved in mitochondrial biogenesis, such as nuclear respiratory factor 1 (NRF-1) and mitochondrial transcription factor A, as well as genes encoding antioxidant superoxide dismutase (SOD1, SOD2) and catalase [138]. Research shows that mice with impaired *NFE2L2* expression have reduced exercise capacity, reduced energy expenditure, reduced mitochondrial volume, and reduced antioxidant activity after exercise training. Furthermore, in muscle cells, ROS and NO influence mitochondrial biogenesis via the NFE2L2/NRF-1 pathway [15]. These findings highlight the critical role of ROS and NO in regulating *NFE2L2* expression, which supports mitochondrial biogenesis and antioxidant defences in skeletal muscle. Dysregulated *NFE2L2* expression impairs exercise performance, energy expenditure, and mitochondrial function, underscoring the importance of this pathway for muscle health and adaptation to physical activity [15].

Meta-analyses carried out by Pearce et al. [139] show that depressive disorders pose a significant challenge to global mental health with increasing prevalence, particularly in developed countries, and increasing incidence among young people, including children [140]. As the leading cause of mental health-related disease burden, depression can be alleviated by physical activity [139]. Preclinical and animal studies that simulate depression-like behaviours provide valuable insights into the underlying mechanisms. Recent research suggests that the Nrf2 pathway, known for its role in oxidative stress and neuroinflammation, may be involved in the regulation of such behaviours [139,140,141,142]. These findings not only confirm the therapeutic potential of physical activity in alleviating depression but also highlight the Nrf2 pathway as a promising target for the development of new interventions.

## 8. Role in Improving Insulin Sensitivity and Reducing Chronic Inflammation

Numerous studies have consistently highlighted the important role of exercise in improving insulin sensitivity, as demonstrated by Bird and Hawley [135], a key aspect of maintaining metabolic health [42]. Exercise improves the ability of cells, particularly muscle cells, to respond effectively to insulin, thereby aiding in the regulation of blood glucose levels, as previously demonstrated by Hall et al. [134]. This effect is mediated by such mechanisms as increased expression of glucose transporters and optimisation of cellular signalling pathways [143]. In addition, physical activity has been shown to reduce chronic inflammation, a hallmark of metabolic dysfunction. Exercise-induced muscle contractions release anti-inflammatory cytokines, such as IL-6, which help to reduce the low-grade inflammation commonly associated with obesity, type 2 diabetes, and cardiovascular diseases [144]. By reducing inflammation, exercise promotes a healthier metabolic environment, enabling the body to better adapt to daily metabolic challenges [145].

Furthermore, substantial evidence supports the notion that preventing obesity, a complex metabolic condition, requires addressing the imbalance between energy intake and expenditure that leads to excessive fat accumulation [4]. Obesity is influenced by a combination of genetic, environmental, and lifestyle factors, including poor diet, physical inactivity, and hormonal imbalances [26,146,147]. Among these factors, Nrf2 signalling plays an important role in the development of obesity. This pathway is essential for regulating oxidative stress and inflammation, both of which are major contributors to obesity-related metabolic disorders. By activating antioxidant defences and modulating metabolic pathways, Nrf2 helps maintain cellular homeostasis, making it an important mediator in mitigating the complications associated with obesity [4].

Research shows that silencing miR-144 (a family of microRNA precursors found in mammals, including humans) in obese mice increases Nrf2 activity by increasing fumarate levels, a critical component for Nrf2 activation, thereby enhancing antioxidant responses [146]. It has been observed that miR-144 suppresses Nrf2 activity in obesity by modulating the TCA cycle enzyme fumarate hydratase (FH), which reduces fumarate availability and impairs antioxidant defences. This study uncovers a novel mechanism by which miR-144 affects the TCA cycle and Nrf2 signalling, suggesting that targeting miR-144 may serve as a potential therapeutic approach to alleviate oxidative stress in obesity-related liver diseases.

In this context, Nrf2 activation emerges as a central factor in enhancing antioxidant defences, reducing chronic inflammation, and improving insulin sensitivity—key processes that are further enhanced by regular physical activity. Exercise naturally stimulates Nrf2, increasing cellular resistance to oxidative stress, while a diet rich in Nrf2-activating compounds complements and enhances this effect [65]. Together, regular physical activity and an antioxidant-rich diet synergistically boost Nrf2 signalling, promoting improved metabolic health and reduced inflammation [5].

## 9. PI3K/Akt Pathway as a Key Regulator of Nrf2 and Insulin Signalling

Numerous studies have highlighted the central role of the PI3K/Akt signalling pathway in regulating cell growth, metabolism, and survival [148]. Activated by signals such as insulin and growth factors, this pathway is critical for glucose uptake, protein synthesis, and cell survival, particularly in such conditions as stroke, myocardial infarction, SARS-CoV-2 infection, and cancer [149]. In addition, the PI3K/Akt signalling pathway interacts with the Nrf2 signalling pathway, amplifying its activation and enhancing cellular antioxidant defences, particularly in physiological and pathophysiological conditions [150].

The activation of the PI3K/Akt pathway has been shown to enhance Nrf2 activity, leading to increased expression of antioxidant enzymes and protective proteins [151]. This interplay between PI3K/Akt and Nrf2 is particularly important for metabolic health, as it not only strengthens the body’s ability to cope with oxidative stress but also supports insulin signalling and glucose metabolism [152]. In metabolic tissues, such as the muscle and liver, the PI3K/Akt pathway improves insulin sensitivity and enhances mitochondrial function, both of which are essential for maintaining metabolic balance [153]. Thus, the synergy between the PI3K/Akt and Nrf2 pathways integrates different cellular processes, enabling a coordinated response to exercise, nutrient intake, and oxidative stress [103].

Previous studies have consistently shown that Nrf2 activation is also triggered by metformin, a commonly prescribed anti-diabetic drug for type 2 diabetes and several other conditions [154]. This activation helps mitigate oxidative stress-induced damage and provides neuroprotection in rodent models of ischaemic stroke, Alzheimer’s disease, Parkinson’s disease, and multiple sclerosis [155]. Meta-analyses carried out by the same authors highlight the significant protective effects of metformin against a wide range of diseases, including cardiovascular diseases, obesity, polycystic ovary syndrome (PCOS), osteoporosis, cancer, neurodegenerative diseases, and even COVID-19.

The protective mechanisms of metformin involve the activation of the LKB1/AMPK pathway, which regulates key intracellular signalling networks, such as NF-κB, PI3K/Akt/mTOR, and Nrf2. Together, these pathways influence inflammation, metabolism, and cell proliferation. In addition, metformin improves immune regulation, promotes autophagy and mitophagy, and exerts epigenetic effects. It also protects against oxidative stress and helps maintain genomic stability, as shown by Ala and Ala [155].

Thus, the PI3K/Akt pathway emerges as a key regulator linking Nrf2 activation with insulin signalling and bridging the oxidative stress response with metabolic regulation [151]. Its dual role highlights the therapeutic potential of targeting this pathway to boost antioxidant defences and improve insulin sensitivity in metabolic disorders.

## 10. Exercise-Induced ROS as a Stimulus for Nrf2 Activation

Nrf2 plays a central role in the molecular mechanisms underlying the metabolic adaptation to exercise [156]. During physical activity, particularly endurance or high-intensity exercise, the body generates ROS as by-products of increased cytokine-mediated mitochondrial activity [144]. As well as causing cellular damage, these ROS act as important signalling molecules [65].

Increased levels of ROS during exercise modify specific cysteine residues on the Keap1 protein, which normally binds to Nrf2 and facilitates its degradation. This modification triggers the release of Nrf2 from Keap1, allowing Nrf2 to translocate to the nucleus [157]. Once in the nucleus, Nrf2 binds to AREs in the DNA and initiates the transcription of genes involved in antioxidant defence, detoxification, and cellular repair mechanisms. This Nrf2 activation serves as a protective response, increasing cellular resistance to oxidative stress and promoting mitochondrial biogenesis—key factors in improving long-term metabolic health and exercise capacity [158]. Through this process, exercise-induced ROS not only signal the need for repair and adaptation but also contributes to improved mitochondrial function and energy production [23,47].

Exercise intensity significantly influences the activation of the Nrf2 pathway and subsequent metabolic adaptations [159]. As reported by Martinez-Canton et al. [160], Nrf2 signalling in skeletal muscle is predominantly activated by ROS-induced redox imbalances, often induced by exercise. Human studies confirm that physical activity enhances Nrf2 signalling, improving muscle function and oxidative resilience [160]. Conversely, Nrf2 knockout mice show reduced muscle strength, increased fatigue, and impaired mitochondrial adaptation to exercise [161]. These findings highlight the critical role of Nrf2 in maintaining muscle performance and supporting mitochondrial function during exercise [160].

Different exercise intensities have different effects on Nrf2 activation in humans, with both moderate and high-intensity exercise affecting oxidative stress responses [159,160]. Moderate-intensity exercise, such as steady-state aerobic exercise, typically results in a moderate increase in Nrf2 activation, which helps to balance oxidative stress and supports cellular antioxidant defences [161]. In contrast, high-intensity exercise, including intense interval or resistance training, induces a more significant Nrf2 response, potentially providing greater protection against cellular damage by upregulating antioxidant enzymes and improving metabolic function [162]. However, the precise relationship between exercise intensity, Nrf2 activation, and long-term health benefits requires further investigation to fully understand the optimal exercise regimes for maximising Nrf2-mediated protective effects. Low- to moderate-intensity exercise induces a controlled increase in ROS, which activates Nrf2 without causing significant cellular damage [163]. This moderate level of ROS production stimulates the upregulation of antioxidant enzymes, mitochondrial biogenesis, and other adaptive mechanisms that help counteract oxidative stress [23,47]. These adaptive responses enhance the body’s ability to neutralise oxidative damage and support cellular function over time [65]. In contrast, high-intensity exercise produces a more pronounced ROS surge which, if not properly regulated, can overwhelm the cell’s antioxidant capacity and lead to oxidative damage [160]. However, even during intense exercise, Nrf2 plays an important role in the body’s adaptive response by further activating antioxidant defences and promoting mitochondrial adaptations. This increased Nrf2 activation helps protect cells from the potentially harmful effects of elevated ROS levels. These findings suggest that regular exposure to exercise-induced oxidative stress, even at high intensities, may improve the body’s long-term ability to cope with oxidative challenges. It also promotes greater metabolic resilience and adaptation, ultimately contributing to improved overall health and fitness [159].

Table 2 presents selected studies that demonstrate how different types and intensities of exercise activate Nrf2 and influence related mechanisms. It highlights how aerobic, resistance, and high-intensity exercise at moderate and high intensities engage Nrf2 pathways to enhance antioxidant responses. These findings highlight the versatility of exercise programmes in modulating oxidative stress and enhancing cellular resilience.

To summarise the data presented in the table, it is important to highlight that exercise-induced Nrf2 activation drives the expression of genes critical for mitigating oxidative damage, reducing inflammation and maintaining metabolic homeostasis. In addition to improving mitochondrial function, Nrf2 activation improves insulin sensitivity by upregulating key proteins involved in glucose uptake and metabolism. This is particularly important in metabolic conditions, such as type 2 diabetes, where insulin resistance is a major challenge. In addition, the upregulation of antioxidant enzymes by Nrf2 activation helps to reduce inflammation, a condition often exacerbated by chronic metabolic disorders. The combined effects of improved mitochondrial function, improved insulin sensitivity, and reduced inflammation facilitate metabolic adaptations that not only support exercise performance but also promote overall metabolic health and resilience to disease.

In summary, the dynamic interaction between exercise intensity and Nrf2 signalling plays a central role in achieving long-term health benefits of regular physical activity, particularly in maintaining metabolic balance and resilience.

## 11. Oxidative Stress During Exercise

During exercise, the body produces ROS as a natural by-product of energy metabolism, particularly in muscle tissue [162]. While excessive ROS production can cause oxidative damage and cellular stress, moderate levels of oxidative stress play an important role in signalling adaptive responses. ROS act as signalling molecules that activate pathways essential for metabolic adaptation, including the upregulation of antioxidant defence systems and mitochondrial biogenesis. This phenomenon, often referred to as “exercise-induced hormesis”, reflects the body’s ability to adapt to mild stress by strengthening its resilience to future stressors [171]. The transient increase in ROS levels during exercise promotes beneficial changes within cells, increasing metabolic flexibility and enabling the body to efficiently switch between energy sources, such as fats and carbohydrates, as needed [1].

Hypoxia preconditioning (HP) refers to the exposure of an organism or tissue to low levels of oxygen (hypoxia) for a short period of time prior to a more prolonged or severe hypoxic event. This preconditioning induces adaptive cellular responses that improve the body’s tolerance to subsequent hypoxic or stressful conditions [172]. In the skeletal muscle, HP has been shown to improve exercise capacity, mitochondrial function, and antioxidant defences, making it a promising strategy for enhancing performance and recovery in athletes or individuals in low-oxygen environments. In addition, HP activates key signalling pathways, including Nrf2, which protect cells from oxidative damage and support metabolic adaptations [173,174].

Several studies have investigated the sources of ROS in contracting muscles and exercise-induced changes in antioxidant enzymes that neutralise superoxide radicals and hydrogen peroxide within muscle fibres [14,137]. These studies also emphasise the role of the Nrf2 pathway, highlighting its critical function in regulating the expression of various antioxidant proteins during exercise. Previous research conducted by the same authors detailed recent advances in understanding how exercise modulates the Nrf2 pathway and its influence on muscle antioxidant defence mechanisms [137].

Wang et al. [174] demonstrated that elite endurance athletes often train in hypoxic or high-altitude conditions to induce stress responses in the skeletal muscle, ultimately enhancing physical performance. Their study investigated the role of Nrf2 in hypoxia preconditioning and its effects on exercise capacity, antioxidant status, and mitochondrial adaptations in the skeletal muscle. The results showed that while Nrf2 deficiency did not affect baseline exercise capacity, it completely negated the performance improvements induced by hypoxia preconditioning. This highlights the essential role of Nrf2 in HP-induced adaptations, including enhanced antioxidant responses and metabolic changes in the skeletal muscle [174].

## 12. Exercise as an Antioxidant Partner

Exercise generates ROS, but dietary antioxidants can play an important role in managing the associated oxidative stress. Antioxidants, which are commonly found in fruits, vegetables, and functional foods, help neutralise excessive ROS and mitigate oxidative damage during the recovery phase following exercise [5]. A review by Done and Traustadóttir [23] highlights that both regular and acute exercise are crucial for activating the Nrf2 pathway, a key regulator of the body’s antioxidant defences. Acute exercise-induced oxidative stress triggers Nrf2 activation and, with repeated bouts of exercise, this leads to the upregulation of cytoprotective genes. As a result, the body becomes more adept at combating oxidative damage, with systemic benefits extending beyond the skeletal muscle to the organs, such as the heart. These findings highlight the critical role of Nrf2 in mediating the health benefits of exercise. The interplay between exercise-induced oxidative stress and dietary antioxidants creates a synergistic effect that supports metabolic adaptation, highlighting the need for a balanced approach to physical activity and diet to optimise metabolic health [175,176].

Irisin, a myokine produced and released by muscle cells during exercise, is involved in numerous metabolic processes [177]. It plays a pivotal role in the regulation of energy expenditure by promoting the browning of white adipose tissue, thereby enhancing fat burning and thermogenesis [178]. In addition, irisin has anti-inflammatory properties, reducing inflammation in such conditions as obesity and sepsis [179]. It also influences macrophage differentiation, promoting an anti-inflammatory phenotype. This makes irisin a promising therapeutic target for inflammatory diseases [168]. A study by Tu et al. [168] showed that irisin promotes M2 macrophage differentiation via activation of the JAK2-STAT6 pathway. This activation enhances the transcription of PPAR-γ and Nrf2, thereby promoting anti-inflammatory and antioxidant responses in macrophages.

Ostrom and Traustadóttir [159] demonstrated that aerobic training effectively improves impaired Nrf2 signalling in older adults, a critical pathway for maintaining redox balance. Their study showed that while older individuals have a reduced Nrf2 response to acute exercise compared to younger adults, 8 weeks of moderate aerobic exercise significantly improved this signalling, specifically by lowering basal Nrf2 levels and increasing the acute response. These findings highlight that regular exercise can help reverse some of the age-related decline in antioxidant defences, although not to the extent seen in younger individuals.

Another study [47] examined the effects of a single session of submaximal aerobic exercise on Nrf2 signalling in both young and older men. The researchers found that although both age groups exhibited increased whole-cell Nrf2 levels following exercise, the nuclear accumulation of Nrf2 and the activation of downstream antioxidant genes (*HMOX1* and *NQO1*) were significantly higher in younger participants, whereas these responses were impaired in older adults. These results highlight the age-related reduction in Nrf2 nuclear import and antioxidant gene expression following exercise, providing new insights into the reduced resistance to oxidative stress in ageing.

Physical activity therefore plays a crucial role in maintaining health at a molecular level, particularly in older adults. Exercise stimulates the body’s production of natural antioxidants, strengthening mitochondria and reducing inflammation. By boosting the body’s antioxidant defences, antioxidants can help reduce inflammation and muscle damage, improving recovery and adaptation to exercise [180]. In addition, antioxidants protect mitochondrial health by protecting mitochondria from oxidative damage, which is essential for maintaining long-term exercise performance and promoting metabolic health [5]. Regular moderate activity, such as jogging, yoga, or swimming, is recommended as the most effective strategy. It is important to tailor the intensity and type of exercise to individual ability and age to avoid overloading the body, which can lead to increased oxidative stress. A balanced approach ensures long-term benefits without unintended risks [176].

## 13. Synergy Between Functional Foods and Exercise

Both functional foods and exercise share common mechanisms that target key aspects of metabolic health, such as oxidative stress, inflammation, and mitochondrial dysfunction. These interventions work synergistically to improve metabolic outcomes by enhancing the body’s ability to adapt to physiological stress [181]. At the molecular level, functional foods rich in such antioxidants as polyphenols and flavonoids help reduce oxidative stress by neutralising ROS- and NO-dependent pathways, while exercise induces controlled oxidative stress that activates adaptive pathways [182]. The summary in Table 3 highlights the role of functional foods and their effects on Nrf2 activation, emphasising their importance in managing oxidative stress and inflammation. It presents selected studies that illustrate the impact of specific functional foods and their bioactive compounds on Nrf2-related pathways in different health conditions. This comprehensive review highlights the potential of dietary interventions to enhance antioxidant defences and promote overall cellular health (Table 3).

Moderate exercise is known to increase ROS production, which activates the Nrf2 pathway and upregulates the body’s endogenous antioxidant defences [187]. When combined, functional foods and exercise create a balanced environment that mitigates oxidative damage and optimises metabolic function. Studies have shown that integrating dietary interventions with exercise improves such biomarkers as blood glucose levels, lipid profiles, and mitochondrial health, highlighting the complementary role of these strategies in enhancing metabolic resilience [188].

The rising incidence of cancer, despite advances in prevention strategies, highlights the importance of biochemical and genetic dysregulation, particularly increased oxidative stress and chronic inflammation as major contributors to multistep carcinogenesis [189]. The transcription factor Nrf2 plays a central role in counteracting these processes by regulating the expression of antioxidant and phase 2 detoxification enzymes, which neutralise free radicals and eliminate carcinogens, respectively [190]. However, effective reduction of oxidative stress and inflammation requires a dual approach: activation of Nrf2 to increase antioxidant enzyme levels and replenishment of dietary and endogenous antioxidants depleted in a high-oxidative environment [67].

The complementary roles of functional foods and exercise in activating Nrf2 highlight their combined potential to enhance antioxidant defences, maintain redox balance, and protect against oxidative stress-related diseases [191]. Functional foods, especially those rich in antioxidants, provide essential substrates that help mitigate oxidative stress generated during exercise [182]. These foods contain bioactive compounds, such as flavonoids, carotenoids, and vitamins, which not only neutralise ROS but also modulate the inflammatory response through oral doses and favourably influence genes associated with chemoprevention, as demonstrated in cellular defence mechanisms, including redox status and detoxification [192]. For example, studies in animal models have shown that such compounds as resveratrol, found in grapes [193], and quercetin, found in apples and onions, can reduce systemic inflammation and oxidative stress [194]. In addition, research has confirmed that sulforaphane is a more potent activator of Nrf2 and induces the expression of cytoprotective genes compared to commonly used phytochemical supplements such as curcumin, silymarin, and resveratrol [192].

On the other hand, exercise triggers adaptive processes, such as mitochondrial biogenesis and upregulation of antioxidant enzyme systems. Physical activity stimulates the AMPK (AMP-activated protein kinase) pathway, which promotes the production of proteins involved in energy metabolism, including antioxidant enzymes [195]. Consumption of antioxidant-rich functional foods provides the body with the necessary tools to counteract exercise-induced oxidative stress, creating a feedback loop that supports both short-term recovery and long-term metabolic adaptation. The prevalence of chronic diseases, e.g., metabolic disorders, hypertension, heart diseases, cancer, and diabetes, highlights the importance of these interventions [196].

As reported by Done and Traustadóttir [23], the molecular mechanisms underlying the synergy between functional foods and exercise show how these factors work together to enhance cellular resilience, promote antioxidant activity, and regulate key signalling pathways, e.g., Nrf2, for improved health outcomes. This synergy is explained by their complementary effects on key molecular pathways [25,197]. For example, exercise-induced oxidative stress activates the Nrf2 pathway, which is essential for cellular defence against oxidative damage. This transcription factor upregulates the expression of antioxidant enzymes, such as superoxide dismutase (SOD) and glutathione peroxidase (GPx), which reduce ROS levels and protect cells from damage [67]. Functional foods rich in antioxidants, such as vitamin C, vitamin E, and polyphenols, further enhance the Nrf2 response, thereby amplifying the antioxidant defence mechanisms activated by exercise. In addition, exercise-induced ROS stimulate mitochondrial biogenesis by activating PGC-1α, which is involved in the production of new mitochondria, as reported by Halling and Pilegaard [198].

Functional foods that promote mitochondrial health, such as those containing CoQ10 and alpha-lipoic acid, may enhance the adaptive response of mitochondria to exercise-induced stress, improving cellular energy production and metabolic efficiency [199]. Specifically, the authors recommend a combination of CoQ10, B vitamins/NADH, L-carnitine, vitamin D, and α-lipoic acid for the treatment of neurodegenerative disorders, including amyotrophic lateral sclerosis, Parkinson’s disease, Alzheimer’s disease, multisystem atrophy, and progressive supranuclear palsy [199]. The antioxidants found in functional foods are critical in supporting these adaptive processes, helping to maintain mitochondrial integrity, reduce inflammation, and improve energy metabolism. In addition, exercise increases the absorption and utilisation of nutrients, facilitating the effective absorption and utilisation of antioxidants from functional foods [200]. This dynamic interaction between antioxidant-rich foods and physical activity leads to improvements in metabolic markers, such as insulin sensitivity, lipid metabolism, and fat oxidation, as discussed earlier in this paper.

Both ROS-dependent and ROS-independent mechanisms have been shown to contribute to Nrf2 activation, with several phytochemicals and antioxidants identified as potential activators, as shown in a study by Prasad [189]. However, despite promising experimental evidence, clinical trials of antioxidants for cancer prevention have produced inconsistent results, likely due to variations in study designs. To achieve more reliable and effective results, future clinical trials should focus on high-risk populations, carefully monitor Nrf2 activation, and use combined strategies that increase both antioxidant enzyme levels and chemical antioxidants.

## 14. Activation of Nrf2 by Natural Bioactive Compounds and Exercise-Induced Oxidative Stress

Numerous studies have highlighted the role of resveratrol, a natural phenolic compound found in grapes, berries, red wine, peanuts, and various other plants, as an antioxidant substance [201,202]. Resveratrol is known for its potent antioxidant and anti-inflammatory properties [203]. It achieves these effects by stimulating the production of antioxidant enzymes, thereby reducing oxidative stress, and by regulating critical nuclear factors, in particular Nrf2 and NF-κB, which play a central role in the inflammation-oxidative stress cycle [204]. These molecular mechanisms underlie its potential therapeutic applications in conditions associated with chronic inflammation and oxidative damage [205,206].

Animal studies have demonstrated significant antioxidant and anti-inflammatory benefits of resveratrol, including its effects on broilers [207], the F1 and transgenic F2 generations of Senescence Accelerated Mouse-Prone (SAMP8) females [208], and a rat model of periodontitis [209]. However, clinical trials in humans have yielded inconsistent results [210]. For example, one study found no significant effect on metabolic or anthropometric parameters other than reductions in weight, BMI, and blood pressure. Furthermore, resveratrol was well tolerated, with no serious adverse effects reported [211]. A study by the authors showed that maternal supplementation with resveratrol in mice could prevent cognitive impairment in offspring by inducing epigenetic changes and modulating key cell signalling pathways [208]. These findings suggest that resveratrol may offer a therapeutic avenue for mitigating cognitive decline, potentially through both direct and inherited epigenetic effects.

Despite promising results from animal studies, the therapeutic effects of resveratrol in humans remain inconclusive, and further research is needed to confirm its efficacy and safety. These findings support the hypothesis that polyphenolic and phytochemical compounds confer health benefits. Resveratrol, along with other polyphenols e.g., curcumin, quercetin, and green tea flavonoids, is widely recognised for its potent antioxidant and anti-inflammatory effects [13,201]. These compounds have been associated with a reduced risk of chronic diseases, including cardiovascular diseases and cancer, primarily through the activation of the Nrf2 pathway [212,213]. This pathway enhances the body’s natural antioxidant defences, reducing oxidative stress and inflammation [67]. By modulating these processes, polyphenolic compounds may promote long-term health and reduce the risk of chronic diseases.

Recent research highlights the therapeutic potential of other bioactive phytochemicals such as berberine, tamarind xyloglucan and sulforaphane. Berberine is known for its anti-diabetic and anti-inflammatory properties [214,215], while tamarind xyloglucan promotes gut health [216]. Sulforaphane, an isothiocyanate found in cruciferous vegetables, plays a key role in detoxification processes and regulates gene expression, in particular by upregulating Nrf2. By activating Nrf2, these compounds help protect cells from damage and strengthen the body’s defences against various diseases, including cancer [192].

In addition, dietary compounds, such as ginger, flaxseed oil, and silymarin, also show remarkable health benefits [13]. Ginger and flaxseed oil contribute to cardiovascular health and have anti-inflammatory effects [217,218], while silymarin supports liver function and protects against toxins [219]. These compounds work synergistically to influence the Nrf2 pathway, boosting antioxidant defences, reducing inflammation, and lowering disease risk. Together, they offer a comprehensive approach to promoting overall health and preventing chronic diseases, emphasising the critical role of diet and natural compounds in disease prevention and management [13].

Mitochondrial function is essential for energy production and overall metabolic health, particularly during exercise [57]. Mitochondrial biogenesis, i.e., a process by which new mitochondria are formed in cells to meet increased energy demands, is closely linked to interactions with PGC-1α, particularly in the context of longevity [220]. There are currently two main perspectives on the regulation of mitochondrial biogenesis: one suggests that Nrf2 directly controls the process [221], while the other proposes that Nrf2 primarily influences antioxidant gene expression, with PGC-1α acting as the sole regulator [220]. Evidence from various studies points to a regulatory loop that integrates the roles of both PGC-1α and Nrf2, suggesting that their interplay is crucial [220]. The key contributors to mitochondrial biogenesis include PGC-1α and Tfam (mitochondrial transcription factor A), both of which are essential for mitochondrial DNA replication, transcription, and activation of metabolic pathways [222].

Exercise is a powerful stimulus for mitochondrial biogenesis, increasing both the number and functionality of mitochondria in cells [223]. In addition, antioxidant-rich foods support mitochondrial health by reducing oxidative stress and promoting the expression of PGC-1α and Tfam [224]. Researchers often measure PGC-1α and Tfam levels to assess the effects of exercise and antioxidant-enriched diets on mitochondrial function and their combined effects on metabolic health [225]. Exercise not only increases antioxidant capacity but also strengthens the body’s long-term ability to cope with oxidative stress [181]. The combined benefits of antioxidant-fortified functional foods and exercise-induced Nrf2 activation improve cellular resilience, metabolic flexibility, and overall health by maintaining redox balance and supporting an efficient response to oxidative challenges [197].

## 15. NF-κB in the Modulation of Inflammatory Processes by Nrf2

The nuclear factor kappa-light-chain-enhancer of activated B cells (NF-κB) is a transcription factor central to the regulation of inflammation, particularly in metabolic tissues [226]. In normal conditions, NF-κB remains inactive in the cytoplasm, as it is bound to inhibitory proteins, such as IκB. However, in response to stressors, e.g., oxidative damage or inflammatory signals, NF-κB becomes activated and translocates to the nucleus, where it drives the expression of pro-inflammatory cytokines [227]. Chronic inflammation in metabolic tissues is a hallmark of such conditions as obesity, insulin resistance, and type 2 diabetes [228].

In contrast, Nrf2 is a critical regulator of the cellular antioxidant response, and emerging evidence suggests that it also influences NF-κB signalling [67]. The activation of Nrf2 suppresses NF-κB activity, thereby reducing the production of pro-inflammatory cytokines in metabolic tissues. This interaction between Nrf2 and NF-κB, as highlighted in a study by Casper [229], is essential for maintaining metabolic homeostasis. By reducing inflammation through Nrf2 activation, it is possible to alleviate the chronic low-grade inflammation associated with metabolic disorders. Thus, Nrf2 not only protects cells from oxidative damage but also plays a key role in regulating inflammatory response, ultimately promoting overall metabolic health [67].

## 16. PI3K/Akt Pathway as a Key Regulator of Nrf2 and Insulin Signalling

The PI3K/Akt signalling pathway is a key regulator of cell growth, metabolism, and survival [230]. It is activated by a variety of stimuli, including insulin and growth factors, and plays a central role in such processes as glucose uptake, protein synthesis, and cell survival. In addition, the PI3K/Akt pathway interacts with the Nrf2 pathway, enhancing its activation and strengthening cellular antioxidant defences [231]. The activation of PI3K/Akt has been shown to upregulate Nrf2 activity, leading to increased expression of antioxidant enzymes and other protective proteins [151].

**Figure 3:**
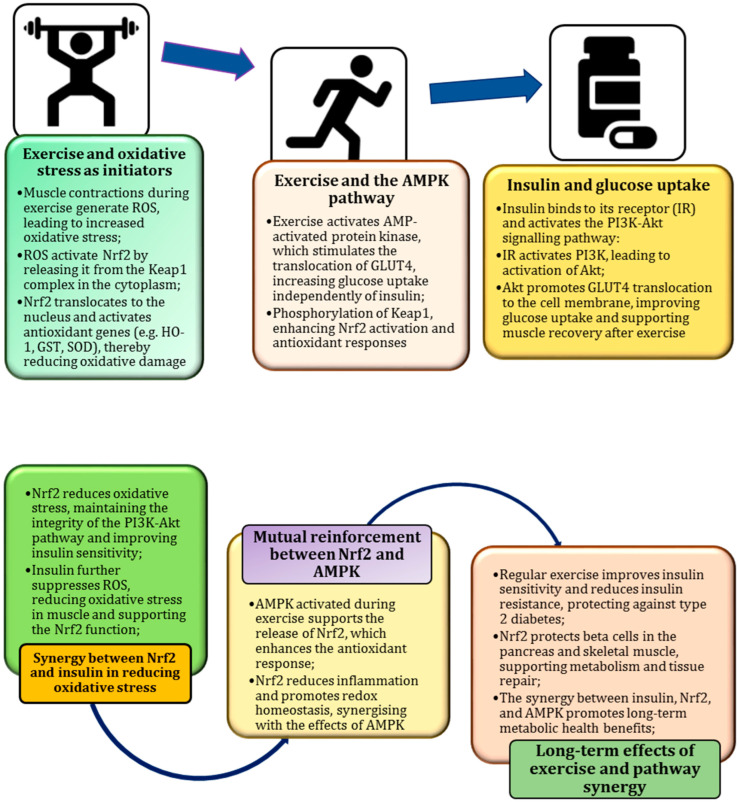
Comprehensive mechanism illustrating the links between exercise, insulin, and Nrf2 from initiation of the process, through exercise, to long-term metabolic benefits.

This interaction between PI3K/Akt and Nrf2, as analysed by Hammad et al. [232], is particularly important for metabolic health. It not only enhances the body’s ability to counteract oxidative stress but also supports insulin signalling and glucose metabolism. Current research shows that in metabolic tissues, such as the muscle and liver, the PI3K/Akt pathway promotes insulin sensitivity and improves mitochondrial function—both of which are essential for maintaining metabolic balance [233]. The interplay between PI3K/Akt and Nrf2 integrates multiple cellular processes, facilitating a coordinated response to exercise, nutrient intake, and oxidative stress [103]. This relationship is illustrated in Figure 3, which provides a visual overview of sequential molecular events and their interconnections, highlighting the mechanisms linking exercise, insulin signalling, and Nrf2 activation.

The interactions between Nrf2 and other signalling pathways, including AMPK, NF-κB, and PI3K/Akt, underscore the complexity of molecular mechanisms governing metabolic health, as highlighted in numerous studies [155,234]. Understanding the crosstalk between these pathways is critical for developing effective therapeutic strategies to improve metabolic health and prevent metabolic diseases.

AMPK has been shown to activate Nrf2, boosting energy metabolism and antioxidant defences, thereby enhancing the body’s ability to adapt to physical activity and energy stress [235]. At the same time, the ability of Nrf2 to inhibit NF-κB activity reduces inflammation in metabolic tissues, further supporting insulin sensitivity and metabolic balance [123]. The PI3K/Akt pathway complements Nrf2 by enhancing insulin signalling, glucose uptake, and mitochondrial function, working synergistically to maintain cellular health [236]. Together, these pathways provide a comprehensive response to oxidative stress, nutrient fluctuations, and exercise, ensuring energy balance, inflammation reduction, and long-term metabolic well-being.

Fasting insulin levels and the homeostasis model assessment of insulin resistance (HOMA-IR) are widely used markers to assess insulin sensitivity and the presence of insulin resistance—key determinants of metabolic health [237]. Insulin resistance plays a central role in the development of such conditions as type 2 diabetes, obesity, and metabolic syndrome [238]. Regular exercise and a diet rich in antioxidants improve insulin sensitivity by improving glucose uptake and utilisation, leading to lower fasting insulin levels and improved HOMA-IR scores [135]. By monitoring insulin levels and calculating HOMA-IR, researchers can assess the combined effects of exercise and functional food interventions on insulin resistance, providing valuable insights into metabolic adaptations [239,240].

## 17. Biomarkers for Assessment of Synergistic Effects

In order to effectively evaluate the combined effects of antioxidant-enriched functional foods and exercise on metabolic health, it is crucial to identify reliable biomarkers that reflect key molecular processes involved in the pathogenesis of “protein conformational diseases”, oxidative stress, and cellular adaptation. These biomarkers provide valuable insights into the synergistic mechanisms by which these interventions improve metabolic function, a topic that has received increasing attention in recent studies. Notable biomarkers include the NAD^+^/NADH ratio [241], the GSH ratio [242], Sestrin2 (SESN2), and 4-HNE adducts [243], each providing unique perspectives on metabolic health and the molecular effects of diet and exercise on metabolic pathways.

In addition, several other biomarkers play an important role in assessing the synergistic effects of exercise and antioxidant-enriched functional foods on metabolic health. These include markers of metabolic function, oxidative stress, and inflammation, which are critical to understanding the broader contributions of diet and physical activity to overall health. Such biomarkers as fasting insulin [240], the homeostasis model assessment of insulin resistance (HOMA-IR) index [244], C-reactive protein, and markers of mitochondrial biogenesis PGC-1α and Tfam [224] improve our understanding of metabolic balance. Their inclusion in assessments deepens insights into how exercise and antioxidant-rich dietary interventions work together to support metabolic health.

Current research highlights the NAD^+^/NADH ratio as a critical marker of energy metabolism and mitochondrial function [245]. NAD^+^ (nicotinamide adenine dinucleotide) is essential for redox reactions and ATP production, serving as a central player in cellular energy metabolism. The balance between NAD^+^ and its reduced form, NADH, reflects the cellular energy state, with a higher NAD^+^/NADH ratio indicating optimal mitochondrial performance and efficient energy production [246].

**Figure 4:**
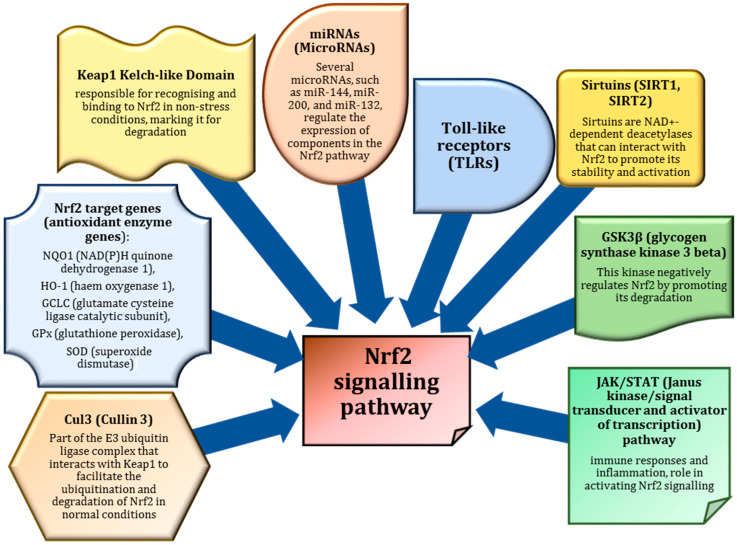
Key elements of the Nrf2 signalling pathway that contribute to its function in cellular defence and redox balance.

Exercise and antioxidant-rich foods positively influence this ratio. For example, Calabrese et al. [241] showed that many phytochemicals exhibit a biphasic dose response in cells, where low doses activate survival pathways and increase the expression of protective protein genes. For example, curcumin activates the Keap1/Nrf2/ARE pathway, while resveratrol enhances the NAD^+^/NADH-sirtuin-1 pathway [247]. Exercise generates ROS, which stimulates sirtuins, i.e., NAD^+^-dependent enzymes that regulate such processes as mitochondrial biogenesis, DNA repair, and energy metabolism [248]. Similarly, functional foods rich in polyphenols and bioactive compounds can increase NAD^+^ availability through e.g., the NAD^+^ salvage pathway [245]. Together, exercise and an antioxidant-rich diet synergistically improve mitochondrial function and energy metabolism, as reflected in a favourable NAD^+^/NADH ratio [249]. Figure 4 illustrates the key elements of the Nrf2 pathway and their role in cellular defence and redox balance.

Glutathione (GSH), a major intracellular antioxidant, plays a critical role in maintaining cellular redox balance and mitigating oxidative stress [250]. Recent studies have shown that GSH activity is enhanced by activation of the Keap1-Nrf2-ARE redox regulatory pathway. This process involves the release of Nrf2, which controls the expression of genes responsible for antioxidant defence, inflammation control, and immune responses. The GSH/GSSG (glutathione peroxidase) ratio serves as a critical marker of cellular antioxidant capacity and overall redox status. A high GSH/GSSG ratio indicates a robust antioxidant defence system capable of neutralising ROS and preventing oxidative damage [251].

Exercise and antioxidant-rich functional foods contribute significantly to maintaining or improving the GSH/GSSG ratio. Physical activity induces mild oxidative stress which, if properly regulated, activates endogenous antioxidant defence mechanisms, including upregulation of GSH synthesis [181]. Similarly, antioxidant-rich foods containing polyphenols, vitamins C and E, and other natural compounds support GSH production and recycling [32]. Together, exercise and dietary antioxidants maintain redox homeostasis, reduce oxidative stress, and promote metabolic health by optimising the GSH/GSSG ratio [252].

Sestrin2 (SESN2) is another critical protein involved in cellular adaptation to metabolic and oxidative stress [253]. SESN2 acts as a key mediator in the regulation of the mTOR (mechanistic target of rapamycin) pathway, which controls cell growth, protein synthesis, and energy balance. It is also integral to autophagy, a process that removes damaged proteins and organelles, promoting cellular rejuvenation and metabolic stability [254]. During exercise, *SESN2* expression is upregulated as part of the adaptive response to metabolic and oxidative stress [255]. Similarly, antioxidant-rich foods containing such compounds as resveratrol, curcumin, and flavonoids can modulate SESN2 levels and enhance its protective effects against metabolic dysfunction and oxidative damage [256]. By facilitating stress adaptation, SESN2 promotes improved mitochondrial function, insulin sensitivity, and metabolic resilience [257]. This makes SESN2 a valuable biomarker for assessing the combined effects of exercise and functional foods on metabolic health [258].

Monitoring lipid peroxidation and oxidative stress using 4-HNE (4-hydroxy-2-nonenal), a highly reactive aldehyde formed during lipid peroxidation, provides valuable insights into cellular damage [259]. Lipid peroxidation occurs when ROS attacks polyunsaturated fatty acids in cell membranes [260]. The formation of 4-HNE adducts with proteins and DNA serves as a key marker of oxidative stress and cellular damage, particularly in such conditions as obesity, diabetes, and cardiovascular diseases, where lipid peroxidation is elevated [261]. Measurement of 4-HNE adducts helps to assess the levels of oxidative stress and lipid membrane damage caused by metabolic challenges [259].

Both exercise and antioxidant-enriched functional foods have been shown to reduce lipid peroxidation and the formation of 4-HNE adducts [262]. While exercise transiently increases ROS production, regular physical activity enhances antioxidant defences and improves mitochondrial efficiency, thereby reducing oxidative damage [181]. Similarly, functional foods rich in antioxidants, such as polyphenols, neutralise ROS and reduce lipid peroxidation [263]. Consequently, monitoring 4-HNE adducts provides a valuable metric for assessing the efficacy of combined exercise and dietary antioxidant interventions in mitigating oxidative damage and improving metabolic health [264].

Another important biomarker, C-reactive protein (CRP), is a well-established indicator of systemic inflammation [265]. Elevated CRP levels are strongly associated with cardiovascular diseases, metabolic syndrome, type 2 diabetes, and chronic low-grade inflammation in metabolic tissues, which play a significant role in the development of these conditions [266]. Both exercise and antioxidant-rich foods help to reduce inflammation by modulating inflammatory pathways. While exercise causes a temporary increase in inflammatory markers, consistent physical activity leads to long-term anti-inflammatory effects [267]. Similarly, antioxidants from functional foods can suppress the production of pro-inflammatory cytokines, thereby reducing CRP levels [25].

In addition, monitoring other inflammatory markers, such as TNF-α (tumour necrosis factor-alpha) and IL-6 (interleukin-6), provides further insight into the anti-inflammatory benefits of these interventions [268]. The incorporation of such biomarkers as fasting insulin, HOMA-IR, CRP, and markers of mitochondrial biogenesis (PGC-1α, Tfam) into metabolic health assessments deepens our understanding of how exercise and antioxidant-enriched functional foods work synergistically to improve metabolic outcomes. These biomarkers complement measures of oxidative stress and antioxidant capacity, providing a comprehensive view of the pathways affected by these interventions [269,270,271].

Recent studies have explored the effects of metabolic reprogramming on cellular functions in response to various stressors, highlighting its critical role in biological processes and cancer [272,273]. Research has demonstrated the influence of metabolic reprogramming on organ protection, inflammation and oxidative stress. For example, studies of pre-operative exercise therapy have shown how metabolic reprogramming in liver cells reduces injury and inflammation [273]. Similarly, heart health studies have shown how Nrf2 activation in heart cells improves glucose metabolism and reduces cardiac dysfunction [274]. These findings highlight the multiple ways in which metabolic reprogramming contributes to cellular resilience and organ protection. These studies highlight the critical role of metabolic reprogramming and Nrf2 activation in improving organ function and protecting against injury. In the context of pre-operative exercise therapy, research shows that four weeks of aerobic exercise significantly reduces liver injury and inflammation caused by ischaemia and reperfusion [275]. This effect is sustained for up to seven days after the end of the exercise programme and is mediated by exercise-induced changes in Kupffer cell metabolism, specifically through activation of high-mobility group box 1 and modulation of itaconate metabolism within the tricarboxylic acid cycle. These changes are Nrf2-dependent, suggesting that exercise not only induces an anti-inflammatory phenotype in liver cells but also enhances cellular defence mechanisms via Nrf2 activation. A similar finding was observed in a study of Nrf2 activation in the heart, which showed that upregulation of the pentose phosphate pathway can improve cardiac function by reducing oxidative stress and supporting metabolic reprogramming [274]. Consequently, these findings highlight the importance of Nrf2 in orchestrating metabolic adaptations that promote cellular resilience, with potential therapeutic implications for both pre-operative care and cardiac health. This is because Nrf2 activation in both liver and heart cells appears to mitigate injury and dysfunction through analogous pathways.

Recent studies have highlighted the potential influence of dietary patterns, particularly those rich in plant-based foods and polyphenol-rich beverages, in promoting mental health and reducing the risk of chronic disease [276,277,278]. This emerging body of research warrants further investigation, particularly with regard to the role of polyphenol-rich alcoholic beverages such as beer and wine. While these beverages have been shown to have potential cardioprotective effects, as demonstrated in studies such as that of Hans et al. [279], their alcohol content may introduce confounding variables in the assessment of mental health outcomes. Therefore, the effect of alcohol consumption on the observed associations with mental health needs to be carefully considered.

In particular, studies investigating the association between consumption of polyphenol-rich beverages and mental health outcomes such as perceived stress, depressive symptoms, and sleep quality, especially in Mediterranean populations, have shown promising results. Research by Micek et al. [276] and Ranneh et al. [278] suggests that moderate consumption of beverages such as coffee and tea is associated with lower levels of perceived stress and depressive symptoms, although the effects on sleep quality remain inconclusive. These findings highlight the need for further research into how polyphenol-rich beverages may contribute to mental health, with a focus on their neuroprotective mechanisms. Beyond mental health, the beneficial effects of polyphenol-rich beverages extend to cardiovascular health, with evidence suggesting that moderate coffee consumption provides protective benefits against atherosclerosis through mechanisms such as prevention of LDL oxidation and promotion of gut microbiota diversity. However, it is important to note that caution should be exercised when considering high coffee consumption, as excessive consumption, particularly due to diterpenes, has been associated with elevated cholesterol levels, posing potential risks to coronary health [277]. These findings highlight the need for balanced consumption, with further research required to better understand the long-term effects of coffee on cardiovascular health and its potential to influence atherosclerosis risk.

**Figure 5:**
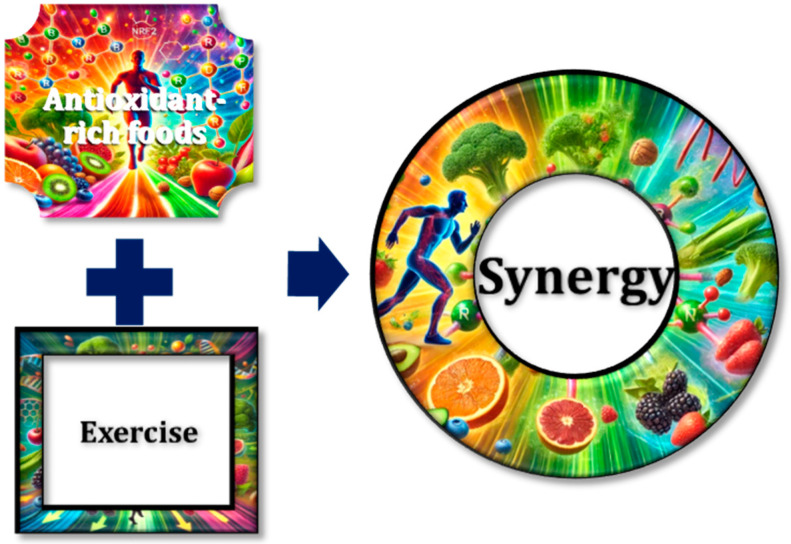
A graphical summary illustrating the synergy between antioxidant-rich foods and exercise in Nrf2 activation.

A graphical summary illustrating the synergy between antioxidant-rich foods and exercise in Nrf2 activation is shown in Figure 5.

While the role of Nrf2 in regulating antioxidant defences and reducing oxidative stress is well documented, the variability of individual responses to Nrf2 activators—both natural and synthetic—remains a critical limitation. Factors such as genetic predisposition, lifestyle differences and baseline metabolic health may influence outcomes, necessitating broader and more diverse clinical trials. Furthermore, while the efficacy of antioxidant-rich diets and exercise in enhancing Nrf2 activity is well documented, further research is needed to validate their efficacy in chronic disease prevention due to potential effects on metabolic pathways. The potential of Nrf2 activators in the treatment of inflammation and diabetes is promising, but precise dosing, safety, and adverse effects require further investigation. Finally, while biomarkers such as the NAD^+^/NADH ratio provide insight into metabolic pathways, it is essential to standardise these measurements to improve reproducibility and reliability. Filling these knowledge gaps would not only improve current understanding but also pave the way for targeted therapeutic applications.

## 18. Conclusions

This review explores the central role of Nrf2 as a key regulator of antioxidant defences required to maintain cellular redox balance. It examines how Nrf2 activation effectively reduces oxidative stress, highlighting its protective role against the initiation and progression of chronic diseases. The review also highlights strategies to enhance Nrf2 activity, such as an antioxidant-rich diet and regular physical activity, either alone or in combination, and demonstrates their significant therapeutic and preventive potential. The review focuses on the most extensively studied therapeutic agents based on Nrf2 activators, including both natural and synthetic compounds. It also discusses the role of Nrf2 in the management of oxidative stress and inflammation, highlighting its therapeutic relevance in such conditions as cardiovascular diseases, diabetes, and neurodegenerative disorders. In addition, the review explains the complementary role of antioxidant-enriched functional foods and physical activity in mitigating oxidative damage and boosting cellular defences. It provides evidence that antioxidant-rich foods provide essential nutrients to activate Nrf2, while regular exercise enhances Nrf2 signalling, thereby boosting the body’s natural antioxidant capacity.

The article also explores the significant impact of physical activity on metabolic health, including its effects on glucose homeostasis, lipid metabolism, and mitochondrial biogenesis. It highlights the role of the PI3K/Akt pathway as a key regulator of both Nrf2 and insulin signalling and introduces such biomarkers as the NAD^+^/NADH ratio, the GSH ratio, Sestrin2 (SESN2), and 4-HNE adducts as valuable tools for understanding the molecular effects of diet and exercise on metabolic pathways. The article concludes by highlighting the importance of Nrf2 activation in enhancing antioxidant defences, reducing chronic inflammation, and improving insulin sensitivity. It explains how physical activity and an antioxidant-rich diet work together to optimise Nrf2 signalling, leading to improved metabolic health and reduced inflammation. In presenting these findings, the article highlights the therapeutic potential of targeting Nrf2 pathways to improve metabolic health and treat metabolic disorders.

## Figures and Tables

**Table 1:** Effects of selected types of diseases on Nrf2 activation and interactions with key signalling pathways.

N	Studies/Disease Models	Key Findings of the Study, Significant Changes in Parameters, Effects of Interventions, and a Summary of the Study	Proposed Mechanism of Action	Sources
1	Hypertension and cardiovascular diseases, chronic diseases	Nrf2 and peroxisome proliferator-activated receptor gamma regulate the expression of detoxification and antioxidant genes and control metabolic and lipid pathways in response to ROS fluctuations	Activation of the Keap1-Nrf2-ARE pathway in response to oxidative stress allows Nrf2 to translocate to the nucleus via regulation of antioxidant gene expression; peroxisome proliferator-activated receptor gamma modulates oxidative stress responses via PI3K/Akt/NOS	[84]
2	Cytoprotection, chemical carcinogenesis, and degenerative diseases	Analysis of the Keap1-CRL complex and SCFβ-TrCP ubiquitin ligase pathways while assessing the role of mTORC1, mTORC2 signalling, PKB/Akt activation, GSK-3-mediated phosphorylation of Nrf2 and ARE-driven gene expression in redox homeostasis and metabolic regulation	Nrf2 is tightly regulated by Keap1-CRL and β-TrCP-SCF complexes in response to oxidative stress and energy-based signals; its activation modulates antioxidant, detoxification, and metabolic pathways via stress-induced or nutrient-dependent pathways, including mTORC1 and PKB/Akt signalling	[85]
3	Models of oxidative stress and inflammation-related diseases, cardiovascular and cardio-metabolic disorders; dependence on antioxidant systems; Nrf2 and HO-1 analysis	The co-ordinated regulation of Nrf2 and HO-1, together with the activation of such antioxidants as superoxide dismutases, catalase, glutathione S-transferase, peroxidase, NAD(P)H quinone oxidoreductase, and thioredoxin, contributes to cytoprotective effects against oxidative stress and inflammation	Activation of Nrf2 involves dissociation from Keap1, nuclear translocation, binding to ARE regions, and subsequent regulation of HO-1 and other antioxidants, resulting in enhanced cellular defence against oxidative damage and inflammation	[86]
4	Erectile dysfunction caused by bilateral cavernous nerve injury, focusing on oxidative stress and inflammation-related nerve damage	Dimethyl fumarate improved erectile function, reduced fibrosis, improved nerve morphology, and increased levels of nNOS, NO, and cGMP, while reducing ROS, 3-NT, NLRP3 inflammasome activation, and markers of DNA damage, with effects dependent on the Nrf2/HO-1 pathway	Dimethyl fumarate activated the Nrf2/HO-1 pathway and protected nerves by decreasing oxidative stress, reducing ROS and NLRP3 inflammasome-mediated pyroptosis, and increasing antioxidant factors, such as SOD, whereas knockdown of Nfe2l2 and Ho-1 attenuated these protective effects	[87]
5	Chronic kidney disease and effects of sulforaphane (400 μg/day for 1 month) on the mRNA expression of *Nrf2*, *NF-κB*, *NQO1*, and markers of oxidative stress in a human model	Chronic kidney disease characterised by redox imbalance, reduced *NrF2* expression, and increased NF-κB levels; sulforaphane increased *Nrf2* and *NQO1* expression and improved serum glucose, phosphate, and triglyceride levels, while reducing LDL-c and total cholesterol levels	Sulforaphane supplementation improves antioxidant systems by increasing *Nrf2* and *NQO1* expression and positively affects serum glucose and phosphate levels, providing a potential therapeutic strategy	[88]
6	Model of endothelial cell injury and atherosclerosis	Herbal and traditional Chinese medicines may act as therapeutic agents by activating the Nrf2/HO-1 pathway to protect vascular endothelial cells from oxidative stress, particularly in the treatment of atherosclerosis.	Protective effects against endothelial cell injury, primarily through the Nrf2/HO-1 pathway, which regulates intracellular defence against oxidative stress	[89]
7	Multiple sclerosis, in particular the relapsing-remitting model; analysis of the protective effect of vitamin D supplementation on DNA repair genes	Vitamin D supplementation for two months significantly altered the expression of DNA repair genes *MYH*, *OGG1*, *MTH1*, and *Nrf2* in multiple sclerosis patients via DNA repair genes and *Nrf2* expression; improved DNA repair efficiency	Vitamin D is likely to modulate the immune system via ARE binding sites in the promoters of *MYH*, *OGG1*, and *MTH1*, potentially enhancing DNA repair processes via Nrf2 activation	[90]
8	*Peritoneal dialysis patients with chronic kidney disease*	Green propolis supplementation reduced plasma tumour necrosis factor-alpha levels and showed a trend towards increased *Nrf2* expression, suggesting its potential anti-inflammatory effect in chronic kidney disease patients on peritoneal dialysis	The use of propolis extract has been shown to reduce inflammation by reducing TNF-α and modulating *Nrf2* expression, which plays a key role in cellular defence against oxidative stress. In addition, no significant effect was observed on nuclear factor-kappa B (NF-κB), a mediator of inflammation	[70]
9	*Supplementation with curcumin (derived from turmeric) for 12 weeks in patients with chronic kidney disease undergoing peritoneal dialysis*	*In patients, oxidative stress was reduced by lowering malondialdehyde levels and uremic toxins, such as p-cresyl sulphate, although no significant changes in inflammatory markers or transcriptional expression, such as Nrf2, HO-1, and NF-κB, were observed*	*The effects of curcumin are mediated through its antioxidant properties, reducing lipid peroxidation, and p-cresyl sulphate; no major changes were seen in inflammatory cytokines (TNF-α, IL-6) or the expression of oxidative stress-related genes (Nrf2, HO-1, NF-κB) in peripheral blood mononuclear cells*	[91]
10	*Patients with type 2 diabetes mellitus and stage 4 chronic kidney disease; comparative analysis of data in both human and mouse models, estimated glomerular filtration rate, metabolic complications, such as oxidative stress, inflammation, and organ damage; bardoxolone methyl, a model of the effects of an Nrf2 activator*	*Bardoxolone methyl increased eGFR, suggesting improved renal function; it caused transient, reversible increases in liver enzymes such as alanine aminotransferase, aspartate aminotransferase, and gamma-glutamyl transferase, without evidence of intrinsic hepatotoxicity*	*Bardoxolone methyl activates Nrf2, resulting in pharmacological induction of ALT and AST isoform mRNA expression in liver and kidney tissues and correlates positively with Nrf2 status, suggesting that the enzyme elevation is related to the Nrf2-mediated transcriptional activity rather than hepatotoxicity*	[92]

**Table 2:** Effects of different types of exercise on Nrf2 activation and interactions with key signalling pathways in experimental models.

N	Study Population/Model—Characteristics of the Study Group or Experimental Model Used	Key Findings of the Study, Significant Changes in Parameters, Impact of Interventions, and a Summary of Implications and Relevance of the Study for the Field	Proposed Mechanism of Action	Sources
1	Zebrafish as an experimental model subjected to a 12-week high-fat diet combined with a swimming exercise intervention; analysis of the antioxidant and protective effects of physical activity on the liver	Swimming reduced lipid accumulation, ameliorated liver damage, and mitigated oxidative stress caused by a high-fat diet; anti-apoptotic effects by increasing the expression of the anti-apoptotic factor bcl-2 and reducing pro-apoptotic genes, such as caspase-3 and bax	Exercise activated the SIRT1/AMPK pathway, improved lipid metabolism, and reduced inflammation via enhanced activation of AKT and Nrf2; upregulation of downstream antioxidant genes, contributing to reduction in ROS and overall antioxidant and protective effects	[164]
2	Adult Wistar rats and a tramadol-treated model; effect of different exercise training protocols (60 days and low, moderate, and high levels) on oxidative stress and testicular endocrine disruption; analysis of recovery of antioxidant and hormonal functions in testicular tissue	Tramadol treatment impaired testicular antioxidant status, testosterone levels, and sperm quality, while low-intensity continuous exercise significantly alleviated these disturbances and improved testicular health	Low-intensity exercise activates the SIRT1/Nrf2 pathway, promoting antioxidant activity and testosterone production through modulation of *miR-126-3p* and *miR-181a* expression	[165]
3	Analysis of effects on Ref1/Nrf2 signalling and mitochondrial H_2_O_2_ production in male ICR/CD-1 mice and models of acute exercise with varying duration	Acute exercise-induced oxidative stress and upregulated Ref1/Nrf2 signalling, which enhanced antioxidant defences and protected cells from oxidative stress during exercise	Acute exercise activated the Ref1/Nrf2 pathway in skeletal muscle, which was associated with increased mitochondrial H_2_O_2_ production and enhanced antioxidant capacity through increased levels of GSH and MnSOD; key role of redox effector factor-1 and Nrf2 signalling	[166]
4	C57BL/6J mouse model; administration of the Nrf2 activator sulforaphane; incremental treadmill exercise to exhaustion under hypoxia; analysis of skeletal muscle markers	Sulforaphane-induced activation of Nrf2 increased the expression of antioxidant genes and MCT1, which improved lactate metabolism and exercise endurance under hypoxia via the lactate/pyruvate ratio by promoting energy production	Sulforaphane pre-treatment improved exercise performance by enhancing antioxidant defences, increasing MCT1 expression, and improving lactate metabolism via activation of Nrf2 as a promising strategy to improve endurance in hypoxic conditions	[167]
5	LPS-induced septic mouse model; RAW264.7 cells in an in vitro model; bone marrow-derived macrophages used to study the effects of the myokine irisin in macrophage polarisation analysis	Irisin induced M2 macrophage differentiation by activating the JAK2-STAT6 pathway, which in turn increased the transcription of PPAR-γ and Nrf2, promoting anti-inflammatory and antioxidant responses in macrophages	Irisin promotes M2 macrophage polarisation through JAK2-STAT6-dependent activation of PPAR-γ and Nrf2; irisin as a promising therapeutic strategy in inflammation and sepsis	[168]
6	Young (18–28 years) and older (≥60 years) participants randomised to an 8-week aerobic exercise training model; Nrf2-related responses assessed before and after a 30-min acute exercise test analysis	Exercise training partially restores impaired Nrf2 signalling in older adults and improves their redox response to exercise, though not to the levels seen in younger people	Aerobic exercise training reduced basal Nrf2 levels while enhancing acute Nrf2 signalling response in older adults via improved expression of antioxidant-related genes	[159]
7	Young (23 ± 1 years) and older (63 ± 1 years) male model; single 30-min session of submaximal aerobic exercise (cycling at 70% VO_2_max) model; assessment of Nrf2 signalling and blood antioxidant responses	Exercise increased total cellular Nrf2 protein levels in both age groups; older adults showed impaired Nrf2 nuclear translocation and suppressed gene expression of key antioxidants, despite similar baseline protein levels	Ageing impairs the Nrf2 nuclear import and downstream antioxidant responses to exercise, highlighting the reduced ability of older adults to counteract oxidative stress compared to younger individuals	[23]
8	Analysis of an exercise model of chronic obstructive pulmonary disease by stabilising Nrf2 and improving mitochondrial function through dissociation of ECH-associated protein 1	Oxidative stress contributes to muscle dysfunction in chronic obstructive pulmonary disease, and exercise helps counteract this by activating Nrf2	Exercise promotes p62 phosphorylation, which competes with Keap1, stabilises Nrf2, and improves muscle function by reducing oxidative stress and improving mitochondrial health	[169]
9	Moderate exercise effects on different exercise parameters (mode, time, intensity) and a model of methamphetamine effects; Nrf2 signalling pathway analysis; dissociation of Keap1 from Nrf2 and mitochondrial function improvement effects	Adequate exercise can attenuate the neurotoxic effects of methamphetamine by activating the Nrf2-mediated endogenous antioxidant pathway, reducing oxidative stress and improving neuronal health	The beneficial effects of exercise are mediated by stabilisation of Nrf2 induced by p62 phosphorylation that competes with Keap1, ultimately improving mitochondrial dynamics and reducing methamphetamine-induced neurodegeneration, oxidative stress, and inflammation	[170]
10	A mouse model of osteoporosis induced by ovariectomy, with Nrf2 gene knockout (*Nfe2l2^−/−^*) mice; analysis of Nrf2 in exercise-induced osteoprotection and its epigenetic regulation; effects of daily 1 h treadmill running on bone mineral density and trabecular microstructure	Exercise, particularly running, could attenuate osteoporosis by reversing epigenetic silencing of *Nrf2*, improving bone mass and microstructure, and normalising the expression of key osteogenic factors, including osteoblast/osteoclast markers and pro-inflammatory cytokines	Repression of Nrf2 by increased levels of DNA methyltransferase, leading to hypermethylation of the *Nrf2* promoter, derepression of *Nrf2*, and activation of downstream antioxidant enzymes, resulting in osteoprotective effects	[156]
11	Review data analysis of the effects of acute and regular exercise on Nrf2 activity and its downstream targets; highlighting the role of oxidative stress in activating Nrf2 signalling	Regular exercise leads to upregulation of Nrf2-mediated antioxidant defences, increasing the body’s ability to counteract oxidative stress-induced damage	Acute exercise induces oxidative stress that activates Nrf2, and, with repeated bouts of regular exercise, this activation results in enhanced cytoprotective gene expression and systemic health benefits	[23]

**Table 3:** Effects of antioxidant-enriched functional foods on Nrf2 activation and key pathway interactions in experimental models.

N	Study Population/Model—Characteristics of the Study Group or Experimental Model Used	Key Findings of the Study, Significant Changes in Parameters, Effects of Interventions, and a Summary of the Implications and Relevance of the Study to the Field	Proposed Mechanism of Action	Sources
1	Effect of soy isoflavone supplementation (55% genistein, 23% daidzein, and 14% glycitein) at a dose of 80 mg/day of isoflavones in a 24-week study in patients with ischaemic cardiomyopathy	Isoflavone therapy improved brachial flow-mediated dilation and reduced triglyceride and LDL-c levels more in females than in males, and reduced serum levels of markers of inflammation and oxidative stress, such as C-reactive protein, 8-isoprostane, malondialdehyde, interleukin-6, and tumour necrosis factor-alpha	The protective effects of soy isoflavones were mediated through up-regulation of Nrf2 and superoxide dismutase, enhancing antioxidant capacity, with no observed effects on oxidation-related molecules in the absence of *Nrf2* expression	[183]
2	Effect of oleic acid (in vitro) in a HepG2 cell model; a rat model of high-fat diet HFD-induced non-alcoholic fatty liver disease (in vivo); study of the therapeutic effects and mechanisms of hesperetin, a citrus derived flavonoid, in a non-alcoholic fatty liver disease model	Hesperetin attenuated hepatic steatosis, oxidative stress, inflammatory infiltration, cytokine secretion via TNF-α, IL-6, and fibrosis; potential of hesperetin as a dietary supplement in the management of non-alcoholic fatty liver disease	Hesperetin reduced ROS production, increased antioxidant activity of SOD, GPx, and HO-1 enzymes, and inhibited inflammatory cytokine secretion (e.g., TNF-α, IL-6), PI3K/AKT-Nrf2 pathway	[16]
3	High-fat diet-fed C57BL/6 mice as an in vivo model; palmitic acid-treated HepG2 cells as an in vitro model to study the effects of chicoric acid	Chicoric acid treatment reduced body weight, adipose tissue mass, hyperglycaemia, dyslipidaemia, hepatic steatosis, oxidative stress, and inflammation in mice fed a high-fat diet; in HepG2 cells, chicoric acid reduced lipid accumulation and oxidative stress; improved gut microbiota composition by increasing the *Firmicutes* to *Bacteroidetes* ratio, promoting a healthier microbial profile	Chicoric acid activates the AMPK/Nrf2 pathway, enhances antioxidant defences, and reduces oxidative stress in liver cells by inhibiting NFκB activation, thereby reducing inflammation and improving lipid metabolism	[184]
4	Effects of epicatechin, a cocoa flavanol, on Nrf2 activation in muscle biopsies from a human model of peripheral arterial disease	Epicatechin increases antioxidants: haem oxygenase-1 (HO-1) and NAD(P)H dehydrogenase [quinone] 1 (NQO1) in the Nrf2 target via reduced myopathy indicators and increased levels of the mitochondrial protein UQCRC2 in muscle	Cocoa flavanols, particularly EPI, enhance antioxidant capacity in PAD by activating Nrf2, improving walking performance, reducing muscle damage, and increasing mitochondrial protein abundance, suggesting that Nrf2 activation is a potential therapeutic target in peripheral artery disease	[185]
5	Oleic acid treated HL-7702 cells in an in vitro steatosis model; high-fat or high-fat and high-cholesterol diet in mouse and rat models; analysis of the effects of gastrodin, a natural compound derived from the root of the plant *Gastrodia elata*	Gastrodin reduced oxidative stress by increasing hepatic superoxide dismutase, decreasing ROS and malondialdehyde (MDA), decreased mRNA levels of proinflammatory cytokines, reduced hepatic steatosis, and improved lipid metabolism by decreasing triglyceride and glucose levels	Gastrodin activates the AMPK/Nrf2 signalling pathway, promoting the phosphorylation and nuclear translocation of Nrf2, which increases the expression of the antioxidant protein haem oxygenase-1 (HO-1), improves lipid metabolism, and reduces inflammation, resulting in suppressed hepatic steatosis and oxidative stress inhibited by compound C	[186]
6.	Ellagic acid, a natural polyphenol, administered at a dose of 180 mg twice daily for 12 weeks to patients in a multiple sclerosis disease model	Ellagic acid supplementation significantly improved depressive symptoms in multiple sclerosis patients by reducing Beck Depression Inventory-II (BDI-II) scores, interferon-gamma (IFN-γ), a pro-inflammatory cytokine, nitric oxide (NO), cortisol, and indoleamine 2,3-dioxygenase (IDO) gene expression, which is involved in tryptophan metabolism and immune regulation, increased brain-derived neurotrophic factor (BDNF) and serotonin	The neuroprotective effects of ellagic acid appear to modulate cortisol, serotonin, BDNF, and IDO gene expression, all of which play a role in mood regulation and neuroprotection. The study did not show a significant effect on Nrf2 levels	[143]
7	Supplementation with Brazilian green propolis extract (400 mg/day) in patients with chronic kidney disease in a peritoneal dialysis model; analysis of inflammatory markers and cytoprotective pathways	Green propolis supplementation significantly reduced plasma tumour necrosis factor alpha (TNF-α) levels and showed a trend towards increased nuclear factor erythroid 2-related factor 2 expression, suggesting its potential anti-inflammatory effect in peritoneal dialysis patients	Propolis extract reduced inflammation by decreasing TNF-α and modulating the expression of Nrf2, a key regulator of cellular defence against oxidative stress, while showing no significant effect on nuclear factor-kappa B (NF-κB), a mediator of inflammation	[70]
8	Effect of sulforaphane extract from cruciferous vegetables administered at 2.5 g/day (containing 1% SFN and 0.5% myrosinase, 2 months) in patients with chronic kidney disease on haemodialysis processes	No significant changes in NRF2 or nuclear factor κB (NF-κB) mRNA expression or in biomarkers of oxidative stress and inflammation (tumour necrosis factor-alpha, interleukin-6) after sulforaphane supplementation	Sulforaphane is known for its potential to activate NRF2 and inhibit NF-κB to reduce oxidative stress and inflammation, but this study showed no significant modulation of these pathways or biomarkers in patients with CKD on haemodialysis, suggesting that the SFN supplementation did not exert the expected antioxidant or anti-inflammatory effects in this cohort	[88]
9	Bioactive phytochemicals, such as resveratrol, curcumin, quercetin, green tea flavonoids, berberine, tamarind xyloglucan, sulforaphane, ginger, flaxseed oil, and silymarin, used to evaluate their effects on health, oxidative stress, and inflammation in human and animal models	The combination of bioactive plant compounds works synergistically to reduce the risk of chronic diseases, such as heart diseases, diabetes, and cancer, by reducing oxidative stress and supporting immune function, potentially improving overall health and preventing the development of diseases	The mechanism of action involves activation of the Nrf2 pathway, which regulates the expression of antioxidant enzymes, reducing oxidative stress and inflammation, protecting cells, and supporting the body’s defence mechanisms	[12,13]
10	Curcumin, a polyphenolic compound derived from the rhizome of *Curcuma longa*; model anti-inflammatory, antioxidant, and anti-apoptotic properties	Curcumin modulates multiple signalling pathways including PI3K, Akt, Nrf2, and STAT3 in the prevention and treatment of diseases, including cancer, metabolic, cardiovascular, and neurological diseases, with a good safety profile	Curcumin exerts its therapeutic effects by modulating key signalling pathways, such as Nrf2 (which regulates antioxidant defences), p38MAPK, and the NLRP3 inflammasome, thereby reducing inflammation, oxidative stress, and apoptosis that underlie several chronic diseases and cancer	[91,110]
